# Pleiotropic roles of elongation factor P in *Bacillus subtilis* physiology revealed by phenotypic and multi-omics analyses

**DOI:** 10.1128/spectrum.03142-25

**Published:** 2026-03-04

**Authors:** Mitsuo Ogura, Yu Kanesaki

**Affiliations:** 1 Institute of Oceanic Research and Development, Tokai University 212250, Shizuoka, Japan; 2 Shizuoka Instrumental Analysis Center, Shizuoka University 13058, Shizuoka, Japan; Rowan University Cooper Medical School, Camden, New Jersey, USA

**Keywords:** transcriptome, proteome, heat tolerance, manganese requirement for growth, motility

## Abstract

Elongation factor P (EF-P) is conserved across the three kingdoms and alleviates ribosome stalling at the XPPX motif of a protein. A genome-wide ribosome-stalling atlas was previously created for the ancestral strain NCIB3610. However, reportedly, ribosome-stalling does not always decrease protein levels. To determine changes in protein levels in the *efp* mutant, we performed a proteomic analysis of the laboratory strain-based *efp* mutant. To evaluate its effects on translation, the amount of protein per mRNA molecule must be determined. Therefore, we performed the RNA-seq experiment. We identified 84 proteins containing the XPPX motif, whose translation required EF-P from 2,187 proteins detected in the proteomic analysis. The *efp*-dependent proteins included major sigma factor SigA, flagella ring component FliY, flagella motor protein MotB, thioredoxin TrxA, and manganese importer MntG. Western blotting revealed a decrease in the RNA polymerase (RNAP) subunits RpoB and RpoC in the *efp* mutant. The decrease in SigA and RNAP levels resulted in altered competition among core RNAP, SigA, and other Sigma factors, leading to a decrease in SigW. A decrease in FliY and MotB caused motility defects, and a decrease in TrxA resulted in a loss of heat tolerance. Moreover, a decrease in MntG altered the manganese requirement for growth at low temperatures. This study reveals the pleiotropic roles of EF-P in *Bacillus subtilis* cellular functions and sheds light on complex interactions between the transcriptome and proteome.

**IMPORTANCE:**

Changes in the proteome have been reported to be consistent with those in the transcriptome. However, a serious change in a gene or protein, such as elongation factor P (EF-P), whose alteration causes pleiotropic effects, leads to proteomic changes that do not fully coincide with the transcriptome. The complex interactions between the transcriptome and proteome result in previously unknown and already known phenotypic changes in *Bacillus subtilis*. Changes in the expression of more than 1,000 genes were observed in the *efp* mutant, whereas changes in only approximately 200 proteins were observed when the same cut-off thresholds were used. This suggests that the robustness of the proteome against severe transcriptomic changes may require largely unknown post-transcriptional regulation.

## INTRODUCTION

Transcription and translation of genes in the genomes of all organisms underlie cellular physiology. Translation is the process through which ribosomes decode the mRNA codon information. After the initiation of translation, subsequent elongation of the nascent peptide chain proceeds at an unequal rate (1–3). In amino acid sequences containing consecutive prolines, the ribosome often stalls because of the unfavorable peptidyl-tRNA geometry, a problem that is alleviated by elongation factor P (EF-P) (4–7). EF-P is conserved in the three domains of life, where it is known as eIF5A in eukaryotes and aIF5A in archaea (8, 9). *efp* is essential in some bacteria; however, its requirement in *Escherichia coli* is conditional, and it is dispensable in *Bacillus subtilis* (10). *B. subtilis* has a protein with an EF-P-like function, YfmR. Simultaneous disruption of *yfmR* and *efp* leads to lethal effects (11). Furthermore, YebC2 was identified as another protein with the ability to resolve ribosome stalling at polyproline tracts in the cells lacking EF-P (12). Although many proteins have consecutive proline residues in their amino acid sequences, ribosome stalling does not occur at all such sites (13, 14). The location of stalling is determined by local context-dependent mechanisms involving the position of the amino acid sequence and the amino acids present upstream and downstream. Therefore, the XPPX motif was proposed as a plausible site (13). Furthermore, not all pauses lead to decreased protein expression (13–16). As expected, ribosome stalling has been observed at several XPPX motifs in genome-wide Ribo-seq analysis using the non-laboratory but wild *B. subtilis* NCIB3610 strain lacking the *efp* gene (17). In the report, transcriptome analysis was also carried out, and a vast fluctuation of gene expression was observed in the *efp* mutant. Moreover, NCIB3610 lacking *efp* showed defects in swarming motility, and this defect was alleviated by many suppressor mutations, including the one that occurred at *fliY* encoding flagella C-ring component (17, 18).

Translation of mRNA, which involves the decoding of codons by ribosomes using aminoacyl-tRNAs. Several enzymes are involved in the maturation of tRNA and rRNA, a required process for translation. One such enzyme is RNase P, which is required for maturation of the 5′-end of tRNA (19). A recent study revealed the involvement of RNase P in mRNA metabolism in *E. coli* (19). The RNA component of the ribozyme RNase P binds to YlxR (RnpM) in *B. subtilis* (20), which was initially identified as a regulator of the glucose response of the extracellular function (ECF) sigma factors SigX and SigM in *B. subtilis* (21, 22). SigX and SigM are involved in resistance against cationic antimicrobial peptides and beta-lactam antibiotics, respectively (21). YlxR is widely conserved in bacteria and regulates the expression of many genes through various modes of action (23–25).

We searched for the regulators of induction of *sigX* by glucose (22, 26), identified EF-P (M. Ogura, unpublished data), characterized it, and provided its first report here. Initial analyses revealed that *efp* is involved in a different regulatory pathway from that involving *ylxR*. We observed that in the *efp* mutant, the amounts of RpoB, RpoC, and SigA decreased, probably because of the presence of the XPPX motif in these proteins. In contrast, in the *ylxR* mutant, the amounts of these proteins did not decrease. To obtain a comprehensive understanding of the effects of *efp* disruption, we performed proteome analysis using isobaric tags for relative and absolute quantification (iTRAQ) coupled with LC-MS/MS (27) and transcriptome analysis using RNA-seq. These methods enabled us to determine the actual changes in protein amounts per amount of mRNA. The part of the inventory of EF-P-dependent proteins with an XPPX motif comprising 84 proteins was confirmed using western blotting. This inventory provided insights into the phenotypes of the *efp* mutant.

## RESULTS

### Varied expression of sigma factors in *efp* mutant

**Fig 1:**
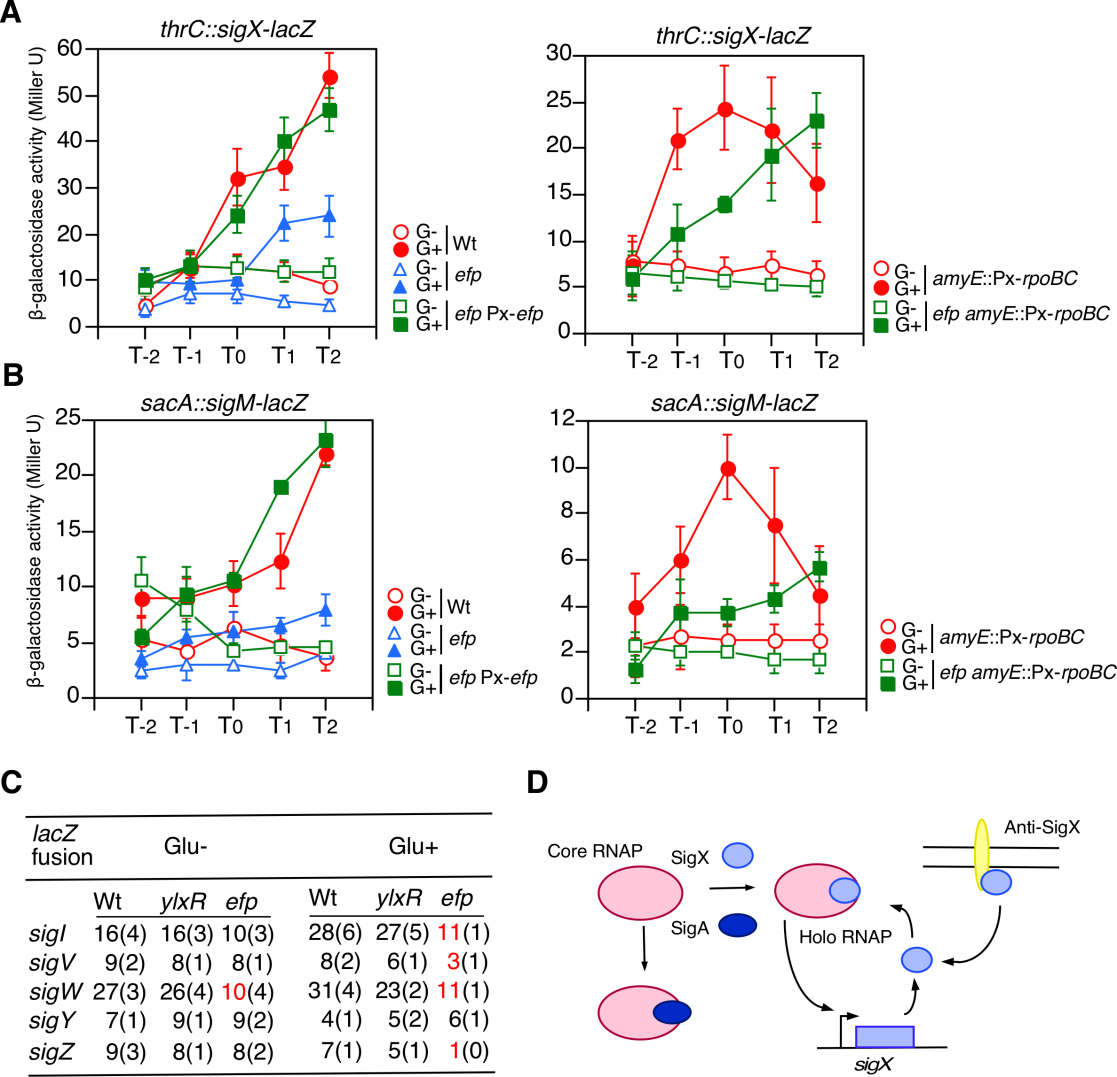
Expression of various sigma factors in the *efp* mutant. β-galactosidase activities are shown in Miller units. Data represent means ± standard deviations of three independent experiments. The *x*-axis represents the growth time in h relative to the end of vegetative growth (T0). Cells were grown in SM. Open symbols, no glucose, and closed symbols with 2% glucose. (**A**) Expression of *sigX-lacZ*. Left: OAM709, wild type; OAM1231, *efp*; OAM1232, *efp* Px-*efp*, 1.8% xylose was added. Right: OAM1233, wild; OAM1234, *efp*, 2% xylose was added. (**B**) Expression of *sigM-lacZ*. Left: OAM1235, wild type; OAM1236, *efp*; OAM1237, *efp* Px-*efp*, 2% xylose was added. Right: OAM1238, wild; OAM1239, *efp*, 3% xylose was added. (**C**) Expression of various sigma genes with or without 2% glucose. Averages of the peak β-Gal activities from three independent experiments are shown with standard deviations. Numbers in red indicate values that decreased compared to those in the wild type. Substrate CPRG was used for *sigV, sigY,* and *sigZ. sigI-lacZ*; MBS78 (wild), OAM1240 (*ylxR*), and OAM1241 (*efp*). *sigV-lacZ*; BSU32 (wild), OAM1243 (*ylxR*), and OAM1244 (*efp*). *sigW-lacZ*; BSU42 (wild), OAM1245 (*ylxR*), and OAM1246 (*efp*). *sigY-lacZ*; BSU35 (wild), OAM1247 (*ylxR*), and OAM1248 (*efp*). *sigZ-lacZ*; BSU36 (wild), OAM1249 (*ylxR*), and OAM1250 (*efp*). (**D**) Schematic representations of competition between RNAP and sigma factors are shown. Box and bent arrows show the open reading frame and promoter, respectively. Ovals represent proteins.

We observed glucose induction (GI) of two ECF sigma factor genes, *sigX* and *sigM* (22). Several regulators of *sigX* GI have been identified using transposon (Tn) insertion mutagenesis, including *ylxR* (22–26, 28–30). During the screening of Tn-inserted regulators, we found that the disruption of *efp* by Tn insertion between the 82nd and 83rd codons resulted in moderate inhibition of *sigX* GI, and that ectopic production of *efp* in this mutant completely restored the GI (left, Fig. 1A). This indicates that *efp* is required for *sigX* GI. A similar trend was observed for *sigM* gene expression (left, Fig. 1B). To investigate whether *efp* is in a feedback loop composed of *ylxR* and the other *sigX* GI regulators (Fig. S1), we examined the effects of *efp* disruption on several sigma factors, including *sigI* and other ECF sigma factors (*sigV, sigW*, *sigY*, and *sigZ*) whose expression was not induced by glucose (Fig. 1C). Disruption of *ylxR* had no effect on the expression of the five sigma genes, whereas disruption of *efp* inhibited *sigW* expression in the absence of glucose and the expression of *sigI, sigV, sigW*, and *sigZ* in the presence of glucose. These results suggest that *efp* affects *sigX/M* expression differently than the *ylxR*-containing feedback regulation system. As *sigX* expression depends on SigX-associated RNA polymerase (RNAP), *sigX* expression is an indicator of the amount of SigX-type RNAP (Fig. 1D). This mechanism also holds true for other sigma factors tested in Fig. 1C (21, 31). Moreover, SigX is trapped by membrane-bound anti-SigX factor when the signal is absent (Fig. 1D) (21). Even under conditions lacking the anti-SigX gene, the effect of *efp* disruption on *sigX* GI was still observed (data not shown), suggesting that *efp* disruption affects SigX protein at the protein-interaction level indirectly. No XPPX motif is present in the amino acid sequences of SigI and the six ECF sigma factors. Thus, the effects on these sigma factors could be caused by the secondary effects of the *efp* disruption.

### Altered SigA and RpoBC amounts in the *efp* mutant

**Fig 2:**
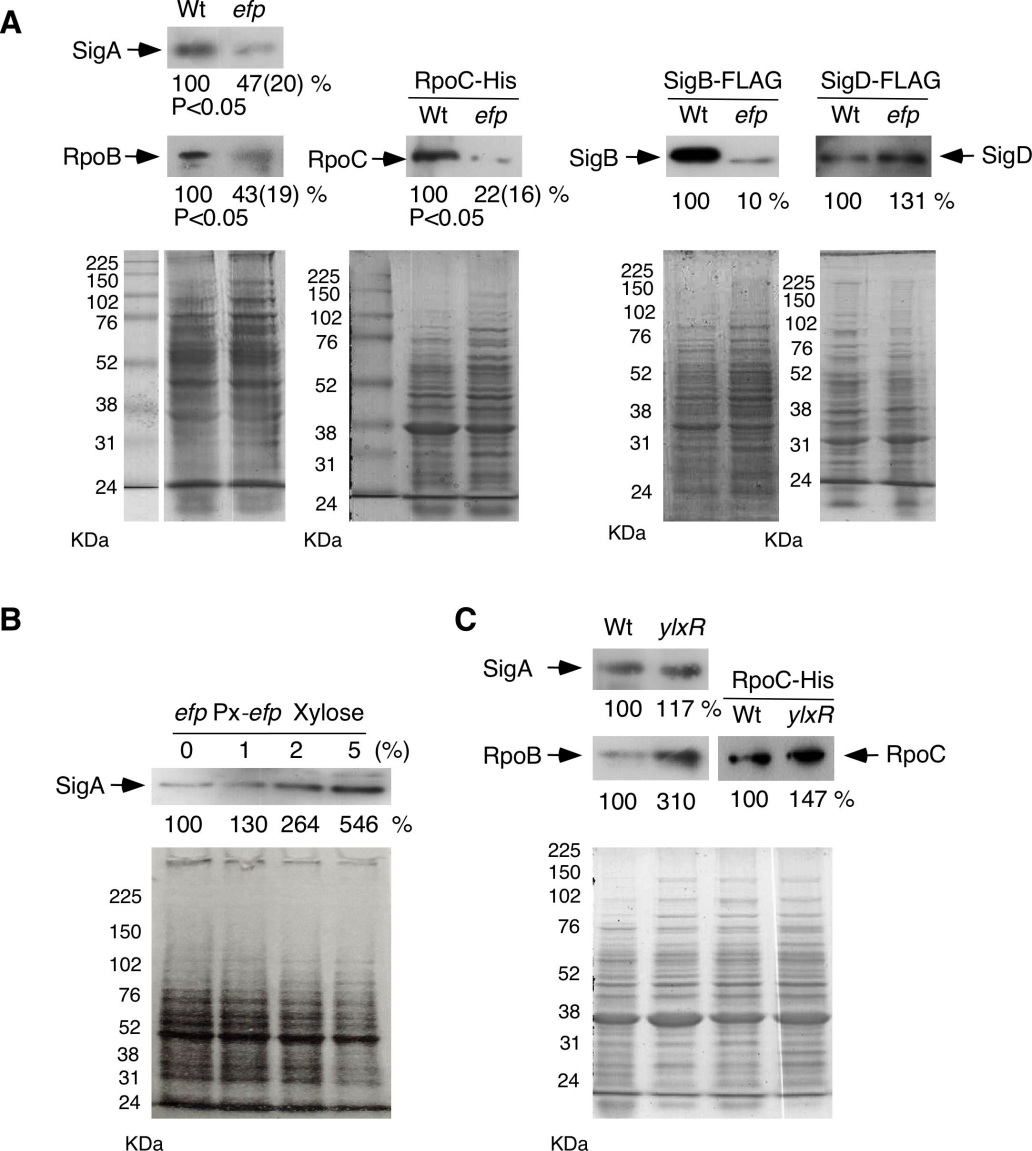
Western blots of RNAP subunits and sigma factors. The procedure and antibodies used are in the Materials and Methods. Cells were grown in SM and harvested at T0. SDS-PAGE is shown as a control. 13% polyacrylamide gels were used. (**A**) Examination of the relative amount of indicated proteins in wild-type and *efp* mutant. Left two panels: strains, 168 (wild), OAM1226 (*efp*), OAM1251 (wild, *rpoC*-His), and OAM1253 (*efp*, *rpoC*-His). Three biologically independent experiments were performed, and standard deviations are shown in parentheses. Significant differences in the changes in band intensities were determined using a non-paired *t*-test, and *P* values are shown. Right two panels: strains, SigB-FLAG (OAM1254, wild; OAM1255, *efp*) and SigD-FLAG (OAM1256, wild; OAM1257, *efp*). (**B**) Effect of *efp* induction on SigA amount. OAM1227 was grown in SM with indicated concentrations of xylose and subject to western analysis. (**C**) Effect of *ylxR* disruption. Strains: 168, OAM816 (*ylxR*), OAM1251 (wild, *rpoC*-His), and OAM1252 (*ylxR*, *rpoC*-His).

The β and β′ subunits of RNAP (RpoB and RpoC) and SigA have possible EF-P-dependent targets for translational regulation because they contain XPPX motifs (EPPT [244th codon] and LPPG [901st codon] for RpoB, IPPE [234th codon] for RpoC, and VPPG [92nd codon] for SigA). If these motifs were dependent on EF-P for translation elongation, *efp* disruption would cause ribosome stalling, leading to downregulation of these proteins. Therefore, it is plausible that a change in stoichiometry between RNAP and sigma factors could lead to altered competition for core RNAP by sigma factors (32, 33) (Fig. 1D). In *B. subtilis* NCIB3610 cells, ribosome stalling has also been observed at these sites (17). Therefore, we examined the cellular levels of these proteins in the *efp* mutant. Western blot analysis confirmed the decreases (Fig. 2A). Furthermore, ectopic induction of the *efp* gene using the xylose-dependent promoter increased SigA protein levels in a dose-dependent manner of xylose (Fig. 2B). This finding reinforces the EF-P-dependent expression of SigA. If alteration of the competition for core RNAP between sigma factors occurs, the inhibition of *sigX* GI could be rescued by the artificial induction of *rpoBC*. Induction of *rpoBC* indeed resulted in the complementation of inhibition of *sigX* GI (right, Fig. 1A). However, when we examined this expectation in *sigM* GI, only partial complementation was observed for unknown reasons (right, Fig. 1B). Finally, we examined the RpoBC and SigA levels in the *ylxR* mutant. RpoC and SigA protein levels were similar in wild-type and *efp* strains, whereas RpoB protein levels increased in the *ylxR* mutant for unknown reasons. These results reinforce the idea that *efp* disruption affects *sigX* GI differently than *ylxR* disruption. The *B. subtilis* genome contains more sigma genes, such as *sigB* and *sigD*. The former has no XPPX motif, whereas the latter has VPPE in its amino acid sequence (179th codon). Therefore, using western blot analysis, we examined whether the levels of these sigma factors changed. In the *efp* mutant, the amount of SigB decreased, whereas that of SigD did not (Fig. 2A). Although SigB protein levels decreased, the ethanol-induced SigB-dependent *ctc-lacZ* expression was similar to that in the wild type (data not shown). This suggests that changes in basal SigB levels do not affect the stress-induced expression of the SigB regulon. Based on these data, we concluded that *efp* disruption caused alteration of competition of sigma factors for core RNAP through stoichiometry changes of these proteins.

### Comparative transcriptome and proteome analyses of *efp* mutant

The above analysis of proteins composed of the core transcription system suggested that the transcriptome of the *efp* mutant changed significantly. The primary target of EF-P is the XPPX motif. However, previous studies have shown that ribosome stalling does not occur at all XPPX motifs, nor does it always lead to decreased protein amount (14, 15). Thus, the actual proteomic changes in the *efp* mutant should be detected. This type of analysis has not yet been performed in *B. subtilis*, despite the identification of its ribosome-stalling profile having been revealed (17). Several comparative proteome analysis methods exist, and we adopted iTRAQ analysis, in which artificial fluctuations caused by technical problems were severely restricted (Fig. S2) (34). In iTRAQ analyses, the observed fluctuations are reported to be generated mainly due to biological fluctuations (34). Therefore, this report recommended that several biologically independent samples be pooled and analyzed in a single iTRAQ analysis to improve experimental accuracy. Based on this report, three biologically independent samples were pooled for each strain and labeled with iTRAQ reagents. The apparent protein amounts should also reflect changes in mRNA levels in addition to translational control. Therefore, we performed RNA-seq analysis using the wild-type laboratory strain 168 and its *efp* mutant, both of which were grown in Schaefer’s sporulation medium at the end of log phase.

### Transcriptome of *efp* mutant

RNA-seq of the three biologically independent samples revealed 478 upregulated and 633 downregulated genes (log2 > |1|) (Table S1). This large fluctuation may be due to alterations in the competition between core RNAP and various sigma factors. Moreover, EF-P deficiency results in fluctuations in several transcriptional regulators that may contribute to this alteration. Notably, the expression of 251 sporulation-related genes was decreased, accounting for 40% of the 633 downregulated genes (Table 1). Information on transcriptomic changes is useful for discriminating between changes in protein levels caused by regulation at the mRNA/transcriptional or protein/translational level.

**TABLE 1:** Summary of RNA-seq and iTRAQ analyses^*a*^

Cellular process	RNA-seq				iTRAQ			
	Increase	Decrease	Total	Total (%)	Increase	Decrease	Total	Total (%)
Cell envelope and cell wall synthesis	27^*b*^	35	62	6^*c*^	0	6	6	3
Transporters	54	39	94	8	1	8	9	4
Homeostasis	4	4	8	0.7	0	6	6	3
Electron transfer and ATP synthesis	19	10	29	3	3	16	19	9
Carbon metabolism	8	22	30	3	3	3	6	3
Amino acids and nitrogen metabolism	29	31	60	5	5	5	10	5
Lipid metabolism	17	7	24	2	2	2	4	2
Nucleotide metabolism	4	7	11	1	0	0	0	0
Additional metabolism	14	32	46	4	2	14	16	7
Detoxification	0	3	3	0.3	0	0	0	0
Genetics	16	9	25	2	1	10	11	6
RNA synthesis and degradation	7	4	11	1	0	1	1	0.5
Protein synthesis, modification, and degradation	52	55	107	10	16	11	27	12
Regulation of gene expression	37	22	58	5	4	8	12	6
Exponential and post-exponential lifestyles	8	12	20	2	1	9	10	5
Sporulation	13	251	264	24	1	18	19	9
Coping with stress	34	21	55	5	0	9	9	4
Miscellaneous lifestyles	2	0	2	0.2	0	0	0	0
Prophage and mobile genetic elements	10	5	15	1	0	6	6	3
Unknown or poorly characterized proteins	123	62	185	17	17	31	48	22

^*a*^ For both analyses, the same cutoff value was adopted: log2 > |1|.

^*b*^ Numbers indicate those of genes for RNA-seq and those of proteins for iTRAQ.

^*c*^ The percentage shows the ratio of the number of genes or proteins contained in each category to the total number detected in the analysis.

### Proteome of *efp* mutant

**Fig 3:**
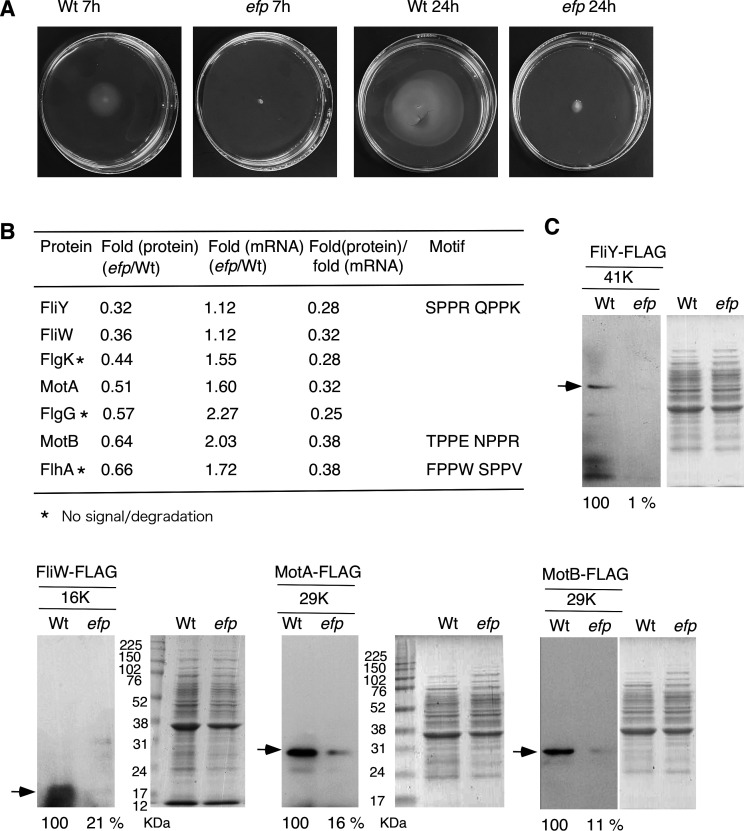
Motility and confirmation of a decrease in flagella-related proteins in *efp* mutant. (**A**) Colony expansion of 168 and OAM1226 (*efp*). The strains were grown in Luria-Bertani (LB) medium overnight and inoculated by using a toothpick onto 0.3% LB soft agar plate, which was left for 5 min on a clean bench. The plates were incubated at 37°C. (**B**) Extent of decreases in flagella-related proteins detected using iTRAQ analysis. Changes in each mRNA amount were detected using RNA-seq. (**C**) Western blot analysis and SDS PAGE. The molecular weight of each FLAG protein is indicated. Strains: *fliY*-FLAG, OAM1258 (wild); OAM1259 (*efp*), *fliW*-FLAG, OAM1260 (wild); OAM1261 (*efp*), *motA*-FLAG, OAM1262 (wild); OAM1263 (*efp*), *motB*-FLAG, OAM1264 (wild); OAM1265 (*efp*). 17% polyacrylamide gels were used.

**Fig 4:**
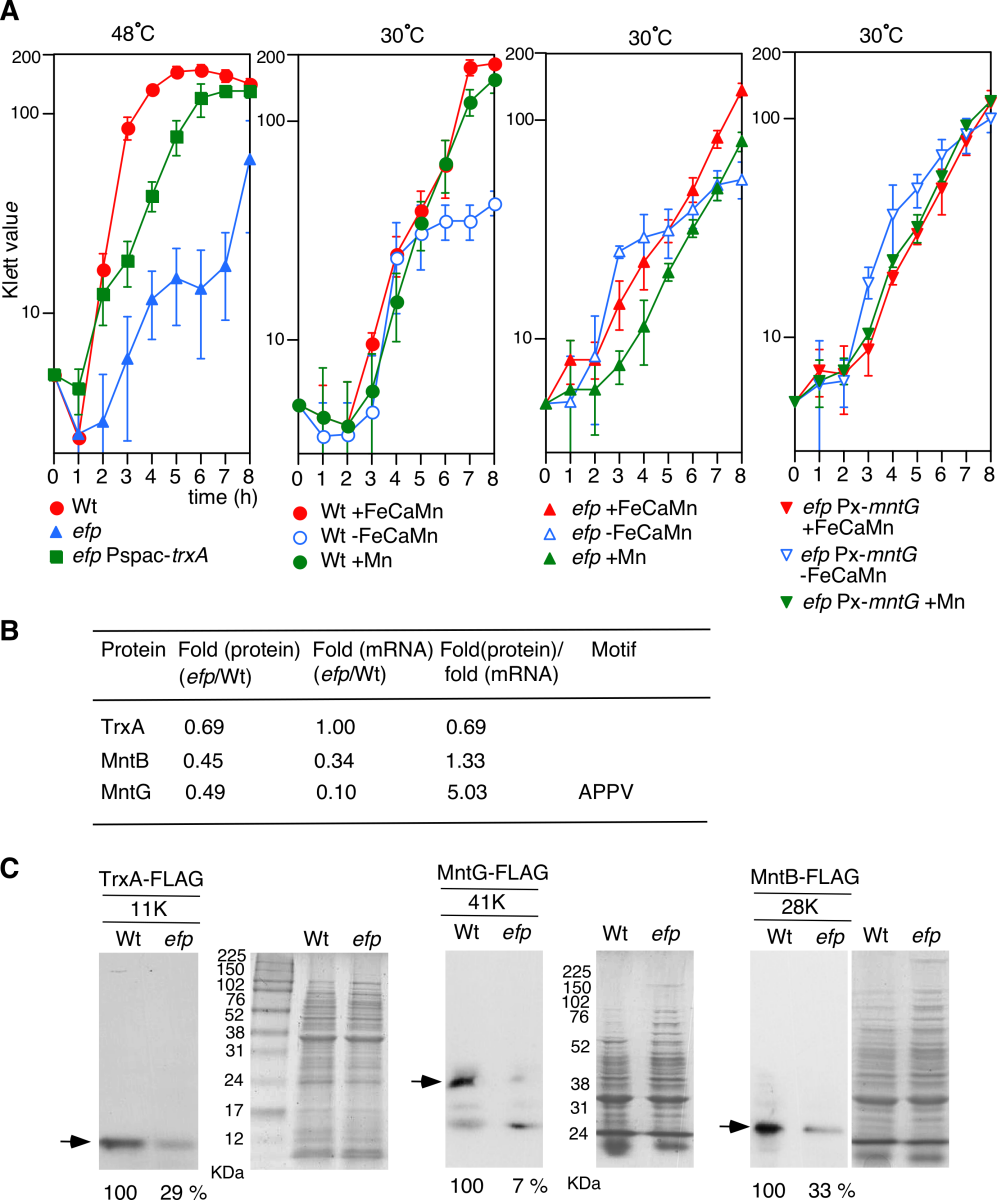
Heat tolerance and manganese requirement in *efp* mutant. (**A**) Cell growth profiles in various mutants. Three biologically independent experiment sets were performed, and standard deviations are shown in the panel. Cell growth was monitored with a Klett calorimeter (Fisher Scientific, MA, USA). Overnight culture grown in LB medium was washed two times with SM medium without three trace metal supplements (final concentration: FeCl_3_, 1 μM; CaCl_2_, 1 mM; MnCl_2_, 10 μM) and inoculated into 4 mL SM medium with or without trace metal supplements in an L-tube. 1% xylose or 0.5 mM IPTG was added, if required. The temperature used in culture is shown over the panel. Strains: 168 (wild), OAM1226 (*efp*), OAM1228 (*efp* Pspac-*trxA*), and OAM1229 (*efp* Pxyl-*mntG*). (**B**) Extent of decreases in proteins detected using iTRAQ analysis. Changes in each mRNA amount were detected using RNA-seq. (**C**) Western analysis and SDS PAGE. The molecular weight of each FLAG protein is indicated. Strains: *trxA*-FLAG, OAM1266 (wild); OAM1267 (*efp*), *mntG*-FLAG, OAM1268 (wild); OAM1269 (*efp*), and *mntB*-FLAG, OAM1270 (wild); OAM1271 (*efp*). 17% and 13% polyacrylamide gels were used for TrxA-FLAG and the rest, respectively.

An iTRAQ analysis was performed using the cells under similar growth conditions to those of the transcriptome analysis. We detected 231 upregulated (>1.5-fold change) and 432 downregulated (<0.7-fold change) proteins. Although this list comprises proteins that function in various cellular physiologies, the proteins involved in protein metabolism were the most altered (Table 1; Tables S2A and S2B). Among the downregulated proteins, 114 contained the XPPX motif. Moreover, the category of downregulated proteins included 109 proteins that were severely downregulated at the mRNA level, suggesting that they were subject to transcriptional rather than translational regulation. Considering transcription-level regulation, 84 proteins were found to be under the control of the protein level and contained the XPPX motif. Therefore, the translation of these proteins would be directly EF-P dependent. Of these 84 proteins, ribosome stalling was observed at the XPPX motifs of 19 proteins in NCIB3610 (17). To confirm the reproducibility of the proteomic analysis, 32 proteins were selected, and a FLAG tag was added to their C-termini. As the amount of SigA, which is often used as a control in western blotting, changed in the *efp* mutant, we used SDS-polyacrylamide electrophoresis of total cellular proteins as a control in this study. Seven proteins showed either no FLAG signal or only degraded products. A decrease in 25 proteins (19 of which carried the XPPX motif) was observed in the *efp* mutant, indicating that those were not false positives in our proteomic analysis (Fig. 3 and 4; Fig. S3). Furthermore, a category comprising 32 proteins with the XPPX motif was identified, in which the apparent protein levels were upregulated. However, considering their increased transcription levels, EF-P-dependent translational downregulation was observed. Of these, ribosome stalling has been observed in the XPPX motifs of eight proteins within NCIB3610 (17) (Table S2A). Finally, we note that YebC2, a protein that functions in the resolution of the polyprolyl sequence (12), was decreased in the *efp* mutant to approximately half of that in the wild-type strain.

### Motility defect in *efp* mutant

Disruption of the *efp* gene in NCIB3610 resulted in the loss of swarming motility (18). This phenotype was rescued by an attenuating ribosome stalling at *fliY*, which encodes a flagella C-ring protein (17). Additionally, previous Ribo-seq analysis in NCIB3610 revealed ribosome stalling in the flagella-related genes, such as *fliF, fliI,* and *motB* (17). Therefore, we examined motility in the *efp* mutant and observed the abolishment of motility (Fig. 3A). Proteomic analysis revealed a decrease in seven flagella-related proteins, three of which contained an XPPX motif (Fig. 3B). As all these proteins showed decreased amounts of protein per mRNA, and ribosome stalling was observed at the XPPX motif of FliY and MotB in NCIB3610 (17), it can be concluded that FliY and MotB are direct targets of EF-P in 168. We attempted to confirm the decrease in protein levels using western blotting, and a decrease in FliY, FliW, MotA, and MotB was confirmed (Fig. 3C). For FlgK, FlgG, and FlhA, no FLAG signal was observed, and only degraded proteins were present. Based on these data, we concluded that the motility defect was caused by the decreased levels of multiple flagella-related proteins.

### Loss of heat tolerance due to decrease in TrxA in *efp* mutant

Many proteins directed by the stress-responsive sigma factor SigB were detected among the EF-P-dependent proteins. Therefore, it was expected that the mutant would have reduced stress resistance. During our search for phenotypes of the *efp* mutant, we observed retarded growth at 48°C, indicating a loss of heat tolerance in the *efp* mutant (left, Fig. 4A). Proteome analysis revealed a decrease in many proteins potentially responsible for the loss of heat tolerance, including TrxA (thioredoxin), which is directed by SigB (Table S2A). TrxA facilitates the reduction of other proteins via cysteine thiol-disulfide exchange (35). TrxA is also known to be heat-inducible and important for preventing protein aggregation during heat stress (35, 36). The decrease in TrxA could be a secondary effect of the *efp* disruption because there was no XPPX motif (Fig. 4B). Decreased TrxA levels were confirmed in the *efp* mutant using western blotting (Fig. 4C). The decrease in TrxA caused a loss of heat tolerance in the *efp* mutant, because the loss of heat tolerance was rescued by the artificial induction of t*rxA* in the *efp* mutant (Fig. 4A).

### Increased manganese requirement for growth in *efp* mutant owing to decreased manganese transporters

We observed a growth defect in the wild-type strain after the middle log phase at 30°C when the medium lacked a trace metal supplement containing iron, manganese, and calcium salts (middle left, Fig. 4A). The addition of iron partially restored the growth, whereas calcium had no effect (data not shown). We found that adding manganese to the medium restored the growth of the wild-type strain to normal levels (Fig. 4A). Cellular manganese mainly functions as an enzymatic cofactor, including superoxide dismutase in *B. subtilis* (37). In this case, the iron requirement was bypassed by the addition of manganese. However, the addition of manganese only partially rescued the growth of the *efp* mutant (middle right, Fig. 4A). Considering that the two manganese transporter proteins were reduced in the *efp* mutant (Fig. 4B and C) (37, 38), this was because manganese was not incorporated into the cells to the extent required for growth to be rescued owing to the decrease in the amount of manganese transporters. Therefore, we examined the possible effects of artificially inducing *mntG* expression in the *efp* mutant. Induction of *mntG* increases intracellular manganese concentrations (38). The strain that overproduced *mntG* with the *efp* disruption was able to grow normally in the absence of iron or manganese (right, Fig. 4A). Furthermore, the addition of manganese did not affect normal growth (Fig. 4A). Therefore, the decrease in MntG expression in the *efp* mutant affected the growth phenotype involving manganese requirements. We noted that MntG carries the APPV motif (171st codon); however, the decrease in protein level was due to a change in transcription rather than translation (Fig. 4B). Therefore, these phenotypes with respect to manganese transport were an indirect consequence of the *efp* mutation. Notably, the *efp* mutant with inducible *mntG* was unstable for reasons that remained unknown.

### Decreases of the Opp system and DefB in *efp* mutant

Several reports have been published regarding sporulation deficiency in the *efp* mutant (39–41). We also observed decreases in the sporulation efficiency of the *efp* mutant (less than 2% of that of the wild-type strain). One of these studies showed that *efp* disruption led to the malfunction of the phosphorylated Spo0A protein, which is a master regulator of sporulation initiation (41). In our proteomic analysis, we observed decreases in OppB and OppC, which carry the LPPE (41st codon) and KPPS (77th codon) motifs, respectively (Table S2A). These two proteins constitute an oligopeptide permease complex required for Spo0A phosphorylation, and defects in this complex are known to lead to sporulation defects (42, 43). Furthermore, DefB, a peptide deformylase essential for sporulation and carrying the LPPT motif (24th codon), was decreased in our proteomic analysis (40, 44) (Table S2A). Decreases in OppC and DefB expression were confirmed using western blotting (#13 and #17, Fig. S3). Thus, we concluded that a decrease in the Opp system and DefB resulted in a sporulation defect and that these would be the consequence of EF-P deficiency. Additionally, a decrease in several proteins required for sporulation that lack the XPPX motif (CotE, PrkA, FacZ, EcsB, AsnO, YhbH, and MinJ) was observed, which would also contribute to the sporulation defect in the *efp* mutant (40).

## DISCUSSION

Using transcriptome and proteome analyses, we identified proteins that are subjected to EF-P-dependent translation regulation. A previous proteome analysis using stable isotopic labeling by amino acids in cell culture (SILAC) in *E. coli* and *Salmonella* serovar *typhimurium* revealed approximately a hundred proteins as EF-P-dependent proteins (14, 15). Different types of proteins were detected among these EF-P-dependent proteins. This suggests that EF-P plays different physiological roles in each bacterium. Indeed, the identified phenotypes in bacteria with *efp* disruption vary; for example, loss of pathogenicity on apples in *Erwinia amylovora* (45), loss of virulence, slowed growth, and increased antibiotic sensitivity in *E. coli* (46), lethality in *Neisseria meningitidis* (47), and increased membrane permeability and infection control through magnesium transporter gene regulation in *S*. serovar *typhimurium* (48, 49).

The genome of *B. subtilis* 168 contains 927 genes with an XPPX motif (50). Of the 165 genes whose translation resulted in weaker attenuation of ribosome stalling in the *efp* mutant than in wild-type NCIB3610, 115 showed translational pausing in their XPPX motifs (17). However, the actual number of proteins showing decreased levels remains unknown. This is important because a phenotype is primarily caused by the actual fluctuation in protein amounts rather than by low translation speed due to *efp* disruption. To determine the proteins that were translated less owing to EF-P loss among those with decreased amounts, data on mRNA fluctuations are required. To confirm this, we performed iTRAQ and comparative RNA-seq using the *efp* mutant. Consequently, 84 proteins that fulfilled these criteria were detected. Moreover, a decrease in the expression of 19 proteins was confirmed using western blotting. Overall, previous ribosome stalling data and/or decreased protein amounts in western blots provide evidence for 30 of the 84 proteins (17). To the best of our knowledge, this type of inventory of the *efp* mutants has not been published. However, to determine whether ribosome stalling occurs at the XPPX motif of these proteins, Ribo-seq analysis of the laboratory strain is required. Our results showed a relatively small number of proteins with the XPPX motif that are under the control of EF-P. In the *efp* mutant, YfmR and YebC2 might rescue ribosome stalling caused by the lack of EF-P, leading to the detection of a small fraction of EF-P-dependent proteins, although YebC2 was reduced because of unknown reasons.

Using our inventory of downregulated proteins in the *efp* mutant, we identified previously unknown *efp* phenotypes and confirmed those already known. Ribosome stalling was observed in the XPPX motifs of 115 proteins in NCIB3610, only 27 of which were found to be subject to EF-P-dependent downregulation in our study. Of note, for constructing the ribosome stalling map, NCIB3610 was grown in Luria-Bertani (LB) medium, and the cells were harvested at a very early log phase (OD_600_ = 0.3–0.4) (17). GFP fusions with *fliP* and *flhP* were downregulated in the NCIB3610-based *efp* mutant grown in minimal salt medium, although neither was detected in the ribosome-stalling atlas (50). Our analysis using laboratory strain 168 and its derivative revealed no change in the levels of these proteins. Furthermore, a decrease in SigA expression was not detected using western blot analysis in the NCIB3610-based strain grown in LB medium (50). NCIB3610 exhibits different behaviors from those of the lab-strain 168, including biofilm formation, surfactin production, high exoprotease production, swarming motility, and low transformability. These behaviors are attributed to genetic differences in the *epsC*, *swrA, sfp,* and *degQ* genes, as well as the presence of the large, low-copy plasmid pLS32 (51, 52). However, these genetic changes alone cannot sufficiently explain the significant alterations in behaviors in EF-P. Therefore, the effects of differences in sampling times and culture media are considered substantial.

In the present study, we observed a decrease in the RpoB, RpoC, and SigA levels in the *efp* mutant. The downregulation of RpoB, RpoC, SigA, and SigB was observed using western blotting, and of SigW using LacZ analysis in the *efp* mutant. Downregulation of SigA and SigW, but not RpoB, RpoC, or SigB, was detected in proteome analysis for unclear reasons. However, we observed downregulation of the RNAP component proteins RpoE (δ subunit), RpoY (ε subunit), and RpoZ (ω subunit), despite the absence of an XPPX motif (Table S2A). RpoE and RpoZ are closely associated with RNAP (53). RpoE is considered an integral RNAP subunit because the ratio of RpoC to RpoE is 1:1 (53). A decrease in RpoE levels was confirmed using western blot (#11, Fig. S3). These observations reinforced a decrease in RNAP holoenzyme levels. If SigB expression had indeed decreased, it is expected that SigB regulon expression would also be reduced. However, the expression of some SigB regulon genes decreased, whereas the expression of others did not, making it difficult to draw conclusions about SigB. While the previous transcriptome study using NCIB3610 revealed the downregulation of the SigD-regulon in the *efp* mutant (17), our transcriptome and western blot experiments did not show such changes. This discrepancy may be caused by different sampling conditions.

The consequences of reduced core RNAP or primary sigma factor in bacteria remain unclear, and only a few cases have been reported. In mycobacteria, an sRNA called Ms1 interacts with the core RNAP, and deletion of Ms1 results in a decrease in RNAP protein levels to approximately 60% through a decrease in their mRNA (54). The Ms1 mutant showed no distinct phenotype other than low tolerance to γ-ray irradiation in the stationary phase. This suggests that core RNAP is fully available for sigma factors in the mutant with low core RNAP levels. In *B. subtilis,* the alterations in the transcriptome were examined when the levels of RpoB, RpoC, and SigA were artificially modulated (55). In the case of a decrease in RpoBC, fewer changes in gene expression were observed than with modulation of SigA, although in both cases the expression of more than a thousand genes was altered (55). Decreasing SigA and core RNAP modulated competition for core RNAP among sigma factors, leading to overexpression of the SigO regulon (55). This indicates that the altered stoichiometry between core RNAP and sigma factors causes altered expression of other sigma regulons, as observed in the *efp* mutant.

Generally, at similar growth rates, the transcriptome and proteome are linked to each other and change in parallel (56, 57). These observations were observed in *E. coli*. However, a serious change in a gene or protein, such as EF-P, whose alteration causes pleiotropic effects, leads to proteomic changes that do not fully coincide with the transcriptome. Changes in the expression of more than one thousand genes were observed in the *efp* mutant, whereas changes in only approximately 200 proteins were observed when the same cut-off thresholds were used. In *B. subtilis,* transcription and translation are decoupled owing to the higher speed of transcription by RNAP on DNA than that of peptide chain elongation by ribosomes on mRNA (58). This characteristic may contribute to the differential alteration of the proteome compared to that of the transcriptome observed in the *efp* mutant. However, the following point should also be considered. Owing to the principle of iTRAQ, where multiple peptides are generated from a single protein and changes are determined by their sum, the dynamic range is compressed, often making the fold changes appear small (59) (Fig. S2).

**Fig 5:**
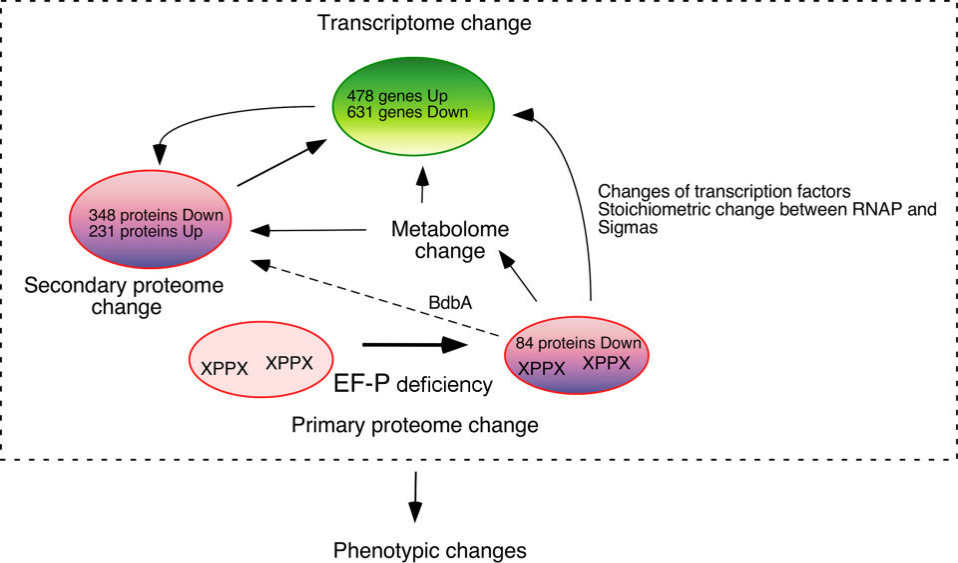
Schematic representation of the changes in the *efp* mutant. Ovals indicate proteome or transcriptome. Transcriptome: in RNA-seq, 4,457 transcripts were detected. DEGs were identified as >2-fold or <0.5-fold changes. Proteome: in iTRAQ, 2,187 proteins were detected. DEGs were identified as >1.5-fold or <0.7-fold changes. Totally, 432 proteins were decreased in the *efp* mutant, and 114 proteins carried the XPPX motif. Of these, 84 proteins exhibited a clear decrease in protein amount per mRNA ratio.

Our study revealed the complicated interactions between the transcriptome and proteome in the *efp* mutant. In the *efp* mutant, 23 proteins expressed from genes with downregulated transcription were upregulated, such as the ribosomal protein genes *rpsE, rpmC, rplX, rpsK*, and *rplW*. In total, 21 ribosomal proteins were detected among the upregulated proteins. It is plausible that enhancing ribosomal proteins could rescue the decreased translation rate caused by ribosome stalling in the *efp* mutant, in addition to YfmR and YebC2. However, a recent study showed that polysome profiles and relative abundance of each ribosome species were similar in the wild-type and *efp* strains (12). Conversely, 56 proteins expressed from genes with upregulated transcription were downregulated. As EF-P functions in translation at the XPPX motif in several proteins, disruption of *efp* initially affects these proteins (Fig. 5). Changes in some of these proteins alter gene transcription, leading to secondary proteomic changes. Moreover, the altered expression of various enzymes leads to changes in the metabolome. This type of change also causes alterations in the transcriptome and secondary changes in the proteome. These holistic changes in the proteome result in phenotypic changes, some of which were analyzed in this study.

It has recently been reported that ribosome stalling was observed at artificial sequences, such as tandem DP repeats in the *B. subtilis efp* mutant, suggesting that EF-P attenuates ribosome stalling at these sites (60). In *E. coli,* EF-P recognizes non-canonical amino acid sequences, such as VPW, IPI, and DPG, and attenuates ribosome stalling (61). In *B. subtilis,* EF-P recognizes non-canonical amino acid sequences, such as IPI, KPG, and DPG (17). These characteristics could explain the decreased levels of proteins lacking an XPPX motif. Furthermore, a recent study in *E. coli* showed that EF-P overproduction resulted in ribosome stalling at the APH motif (61). This suggests that EF-P may have another role: slowing down translation to allow sufficient time for protein domain folding. This observation could explain the increased expression of proteins in the *efp* mutant. Approximately 80% of the increased proteins indeed comprise proteins that have undergone no significant transcriptional change. These suggest that EF-P may play a direct role in the translation of these types of proteins.

In this study, we propose an inventory of the initial and secondary EF-P candidate targets in *B. subtilis*. However, not all the proteins were confirmed using other methods, including western blotting. On the basis of this inventory, we identified previously unknown phenotypes caused by *efp* disruption. Therefore, although this inventory may include false positives and exclude false negatives, it provides a basis for further research.

## MATERIALS AND METHODS

### Strains, media, plasmid, and PCR

*B. subtilis* strains and plasmids used in this study are listed in Table S3. Plasmid construction is described in the Supplementary methods. One-step competence medium (62), Schaeffer’s sporulation medium (SM) (63), Antibiotic III medium (BD Difco, MD, USA), and Luria-Bertani Lenox (LB) medium (BD Difco) were used. The antibiotic concentrations have been previously described (64). Synthetic oligonucleotides were purchased from Tsukuba Oligo Service (Ibaraki, Japan) and are listed in Table S4. PrimeSTAR MAX DNA polymerase (Takara Co., Shiga, Japan) was used for PCR.

### β-galactosidase analysis

Growth conditions and methods for β-galactosidase analysis have been described previously (22). The cells were grown in SM. Growth was monitored and expressed relative to T0, the end of log phase. Furthermore, the highly sensitive substrate chlorophenol red β-D-galactopyranoside (CPRG, Roche, Germany) and 2-nitrophenyl-β-D-galactopyranoside (ONPG, Wako-Fuji, Japan) were used.

### Western blot analysis

Cells were grown in 50 mL SM at 37°C and harvested at the end of log phase. Cell lysis and western blotting were performed as described previously (29, 65). The monoclonal mouse anti-FLAG antibody was purchased from ProteinTech (Rosemont, IL, USA). Monoclonal mouse anti-His tag antibody (clone: OGHis) was purchased from MBL (Tokyo, Japan). Anti*-E. coli* RNA polymerase beta (clone: 8RB13) was purchased from BioLegend (San Diego, CA, USA). Polyclonal rabbit anti-SigA antibody and antigen detection were performed as described previously (22, 29). Full-range rainbow molecular weight marker was purchased from Sigma-Aldrich (Darmstadt, Germany).

### RNA isolation and RNA-seq analysis

*B. subtilis* wild-type (168) and *efp* (OAM1226) strains were grown in 50 mL of SM at 37°C, and 4 mL of culture were sampled at the end of log phase. Three independent cultures were used for each strain. RNA isolation and DNA removal were performed using the RNeasy Mini Kit (Qiagen, MD, USA) according to the manufacturer’s instructions. Ribosomal RNA elimination and cDNA library construction were performed using the NEBNext rRNA Depletion Kit (Bacteria) and TruSeq Stranded mRNA Library Prep Kit (Illumina, CA, USA) for 1 μg of total RNA, according to the manufacturer’s protocol. The detailed sequencing procedure is provided in the Supplementary methods.

### Proteome analysis

Strains were grown in 50 mL SM. For each strain, three independent cultures were prepared and harvested at the end of log phase, where Klett values were very similar (differences were less than 5%), and the independent cultures were mixed, washed three times with Tris-buffered saline (pH, 7.5), resuspended in 8 M urea containing 1 mM phenylmethyl sulfonyl fluoride, and lysed with French Pressure cell. After centrifugation (14,000 rpm for 5 min), the supernatant was immediately frozen, and the samples were sent to Creative Proteomics (NY, USA) for iTRAQ analysis. The detailed procedure is provided in the Supplementary methods.

### Detection of XPPX motif

The motif search was previously performed and presented in Table S3 in reference 50. Based on the data, we carried out manual curation.

## Data Availability

Original sequence reads were deposited in the DRA (DDBJ Sequence Read Archive) database, accession numbers: DRR748488–DRR748493. Detailed experimental conditions and raw mass spectra data are available in jPOST (66). Accession numbers are PXD064716 for ProteomeXchange and JPST003850 for jPOST.

## SUPPLEMENTAL MATERIAL

Supplemental materials spectrum.03142-25-s0001.pdf Supplemental methods, Tables S3 and S4, and Figures S1 to S3.

Table S1 spectrum.03142-25-s0002.xlsx RNA-seq analysis.

Table S2A spectrum.03142-25-s0003.xlsx iTRAQ analysis (Decreased proteins).

Table S2B spectrum.03142-25-s0004.xlsx iTRAQ analysis (Increased proteins).

ASM does not own the copyrights to Supplemental Material that may be linked to, or accessed through, an article. The authors have granted ASM a non-exclusive, world-wide license to publish the Supplemental Material files. Please contact the corresponding author directly for reuse.
