# Supplementary material for: Pleiotropic roles of elongation factor P in Bacillus subtilis physiology revealed by phenotypic and multi-omics analyses
Source: Microbiol Spectr. 2026 Mar 4;14(4):e03142-25. doi: 10.1128/spectrum.03142-25 (PMC13055313; doi:10.1128/spectrum.03142-25)
Supplement: Supplemental materials — Supplemental methods, Tables S3 and S4, and Figures S1 to S3. [file spectrum.03142-25-s0001.pdf]

Pleiotropic roles of elongation factor P (EF-P) in *Bacillus subtilis* physiology revealed by phenotypic and multi-omics analyses

Mitsuo Ogura and Yu Kanesaki

Supplementary Methods

Plasmid construction

To construct each plasmid, PCR products amplified using a specific oligonucleotide pair were digested with the specific restriction enzymes and cloned into the vector digested with the same restriction enzymes. Oligonucleotides, restriction enzymes, and vectors used in this study are listed in Table S4. For pYY1, no restriction enzymes were used, instead an in-fusion method was employed. As shown in Table S3, pSac-Em-sigM is a pSac-Km (1) derivative carrying an erythromycin resistance gene and *thrC*::*sigM-lacZ* from pMT6 (2).

RNA sequencing

The cDNA library was sequenced on an Illumina sequencing platform (Illumina MiSeq), and 2 x 150 bp paired-end reads were generated using NovaSeq 600 S4 Reagent Kit. Adapter sequences in each read were removed using CLC Genomics Workbench ver.11.0 software (Qiagen, Germany). The cleaned read data were mapped to the reference genome (accession number: AL009126.3). The mapping parameters were as follows: mismatch cost, 2; insertion cost, 3; deletion cost, 3; length fraction, 0.8; and similarity fraction, 0.9. Differentially expressed genes of each condition and control were identified with significant thresholds of a fold-change ≥ |2| and false discovery rate adjusted p-value (q-value) < 0.05 obtained by a generalized linear model approach using the CLC Genomics Workbench built-in tools Edge Test (EdgeR v3.4.0).

Proteome analysis

Protein digestion: 20 µL of the Dissolution Buffer from the kit (iTRAQ-8plex Isobaric Label Reagent Set, Sigma-Aldrich) was added to each of two sample tubes (each containing 60 µg of protein). 1 µL of the denaturant from the kit was added and vortexed; and 2 µL reducing reagent was added to each sample tube. After mixing, the samples were incubated at 60°C for 1 h; 1 µL cysteine blocking reagent was added to each tube. After vortexing and centrifugation, the tubes were incubated at room temperature for 10 min. Further, 10 µL trypsin solution was added. After vortex and spin, the tubes were incubated at 37°C overnight. **2.** Peptide labelling: 50 µL of isopropanol was added to each room-temperature vial of iTRAQ Reagent-8plex and 5 µL of Dissolution Buffer was added to adjust the pH to 7.5 to 8.5. The tubes were incubated at room temperature for 2 h, and combined into single tube. **3.** Fraction of the sample: Fractionation of the labeled peptide mixture with 16 components using HPLC. Zorvax80 Å (Aligient tech, CA, USA), column: C18 (4.6 x 250mm, 5 µM). Mobile phases A: water; B: 4-85% acetonitrile. The total flow rate was set to 1 nL/min. **4.** Nano LC: Nanoflow UPLC: Ultimate 3000 nano UHPLC system (Thermo Fisher Scientific, USA). Nanocolumn: trapping column (PepMap C18, 100Å, 100 µm × 2 cm, 5 µm) and an analytical column (PepMap C18, 100Å, 75 µm × 50 cm, 2 µm). Loaded sample volume: 1 µg. Mobile phase: A: 0.1% formic acid in water; B: 0.1% formic acid in 80% acetonitrile. Total flow rate: 250 nL/min. LC linear gradient: 2-8% buffer B in 3 min, 8-20% buffer B in 30 min, from 20% to 40% buffer B in 23 min, and 40-90% buffer B in 4 min. **5.** Mass spectrometer. For iTRAQ-labeled samples, a full scan was performed at 350-1650 m/z at a resolution of 120000 at 200 Th, and the automatic gain control target for the full scan was set to 3e6. The MS/MS scan was operated in the top 15 modes using the following settings: resolution 30000 at 200 Th; automatic gain control target 1e5; normalized collision energy at 32%; isolation window of 1.2 Th; charge sate exclusion: unassigned, 1, > 6; dynamic exclusion 40 s. **6.** Data analysis: Raw MS files were analyzed and searched against *Bacillus subtilis* protein database based on the sample species using MaxQuant (1.6.2.14). The parameters were set as follows: the protein modifications were carbamidomethylation (C) (fixed), oxidation (M) (variable), itraq-8plex; the enzyme specificity was set to trypsin; the maximum missed cleavages were set to 10 ppm, and the MS/MS tolerance was 0.6 Da.

Supplementary References

1. Middleton R, Hofmeister A. 2004. New shuttle vectors for ectopic insertion of genes into *Bacillus subtilis*. Plasmid 51:238-245.

2. Matsuoka S, Seki T, Matsumoto K, Hara H. 2016. Suppression of abnormal morphology and extracytoplasmic function sigma activity in *Bacillus subtilis ugtP* mutant cells by expression of heterologous glucolipid synthases from *Acholeplasma laidlawii*. Biosci Biotechnol Biochem 80:2325-2333.

3. Ogura M, Kanesaki Y. 2018. Newly identified nucleoid-associated-like protein YlxR regulates metabolic gene expression in *Bacillus subtilis*. mSphere 3:e00501-18.

4. Ogura M, Matsutani M, Asai K, Suzuki M. 2023. Glucose controls manganese homeostasis through transcription factors regulating known and newly identified manganese transporter genes in *Bacillus subtilis*. J Biol Chem 299:105069.

5. Ogura M, Asai K. 2016. Glucose induces ECF sigma factor genes, *sigX* and *sigM*, independent of cognate anti-sigma factors through acetylation of CshA in *Bacillus subtilis*. Front Microbiol 7:1918.

6. Matsuoka S, Shimizu Y, Nobe K, Matsumoto K, Asai K, Hara H. 2022. Glucolipids and lipoteichoic acids affect the activity of SigI, an alternative sigma factor, and WalKR, an essential two-component system, in *Bacillus subtilis*. Genes Cells 27:77-92.

7. Asai K, Yamaguchi H, Kang CM, Yoshida K, Fujita Y, Sadaie Y. 2003. DNA microarray analysis of *Bacillus subtilis* sigma factors of extracytoplasmic function family. FEMS Microbiol Lett 220:155-160.

8. Kosono S, Asai K, Sadaie Y, Kudo T. 2004. Altered gene expression in the transition phase by disruption of a Na+/H+ antiporter gene (*shaA*) in *Bacillus subtilis.* FEMS Microbiol Lett 232:93-99.

9. Yano K, Mien YL, Sadaie Y, Asai K. 2011. *Bacillus subtilis* RNA polymerase incorporates digoxigenin-labeled nucleotide in vitro. J Gen Appl Microbiol 57:153-157.

10. Hori K, Kaneko M, Tanji Y, Xing XH, Unno H. 2002. Construction of self-disruptive *Bacillus megaterium* in response to substrate exhaustion for polyhydroxybutyrate production. Appl Microbiol Biotechnol 59:211-216.

11. Guérout-Fleury AM, Frandsen N, Stragier P. 1996. Plasmids for ectopic integration in *Bacillus subtilis*. Gene 180:57-61.

12. Yamamoto H, Kurosawa S, Sekiguchi J. 2003. Localization of the vegetative cell wall hydrolases LytC, LytE, and LytF on the *Bacillus subtilis* cell surface and stability of these enzymes to cell wall-bound or extracellular proteases. J Bacteriol 185:6666-6677.

13. Delumeau O, Lecointe F, Muntel J, Guillot A, Guédon E, Monnet V, *et al*. 2011. The dynamic protein partnership of RNA polymerase in *Bacillus subtilis*. Proteomics 11: 2992-3001.

14. Ogura M, Sato T. Abe K. 2019. YlxR, which is involved in glucose-responsive metabolic changes, regulates expression of *tsaD* for protein quality control of pyruvate dehydrogenase. Front Microbiol 10:923.

15. Hemm L, Miucci A, Riediger M, Tholen S, Kraus A, Georg J, Schilling O, Hess WR. Interactors and effects of overexpressing YlxR/RpnM, a conserved RNA binding protein in *cyanobacteria*. RNA Biol 21:1308-1326.

16. Ogura M. 2020. Glucose-mediated protein arginine phosphorylation/dephosphorylation regulates *ylxR* encoding nucleoid-associated protein and cell growth in *Bacillus subtilis*. Front Microbiol 11:2382.

Supplementary figure legends

Fig. S1. Schemes of glucose-response and feedback regulation including *ylxR*. Arrows and T-bars indicate activation and inhibition, respectively. The dotted line represents the multi-step reaction. An association between CshA and RNAP (13), glucose-stimulated CshA acetylation, and CshA-dependent P*ylxS* expression (5) have been observed. P*ylxS* regulation drives expression of YlxR, which regulates the transcription of *tsaEBD* through YlxR-binding to the promoter of *tsaEBD* (14). The *tsaEBD* products are assembled into tRNA modification enzyme that regulates the translation of pyruvate dehydrogenase (PDH) (14). Pyruvate dehydrogenase provides acetyl-CoA, an acetyl moiety source for CshA acetylation. Acetylated RNAP may alter its affinity for SigX and SigM. Recently, in cyanobacteria, YlxR-binding to RNAP was reported, which may cause a change in the affinity of RNAP to a specific sigma factor (15). Glucose enhances cellular manganese concentrations, resulting in stimulation of expression of *ywlE* (4). *ywlE* encodes a protein arginine phosphatase that protects PDH and TsaD from ClpCP-dependent protein degradation (16).

Fig. S2. Principle of iTRAQ analysis. Numbers in parenthesis indicate molecular weight.

Fig. S3. Western blot analysis using FLAG proteins. Examination of the relative amount of indicated proteins in wild type and *efp* disruptant. 17% and 13% polyacrylamide gels were used. The order of the panel arrangement followed the degree of decrease in protein levels observed in iTRAQ analysis. Molecular weight of each FLAG protein is indicated. Strains. *yfmS*-FLAG, OAM1272 (wild); OAM1273 (*efp*), *yvmA*-FLAG, OAM1274 (wild); OAM1275 (*efp*), *cypX*-FLAG, OAM1276 (wild); OAM1277 (*efp*), *yuzD*-FLAG, OAM1278 (wild); OAM1279 (*efp*), *narH*-FLAG, OAM1280 (wild); OAM1281 (*efp*), *albF*-FLAG, OAM1282 (wild); OAM1283 (*efp*), *pnbA*-FLAG, OAM1284 (wild); OAM1285 (*efp*), *yvcK*-FLAG, OAM1286 (wild); OAM1287 (*efp*), *yscB*-FLAG, OAM1288 (wild); OAM1289 (*efp*), *nasF*-FLAG, OAM1290 (wild); OAM1291 (*efp*), *rpoE*-

FLAG, OAM1292 (wild); OAM1293 (*efp*), *csaA*-FLAG, OAM1294 (wild); OAM1295 (*efp*), *defB*-FLAG, OAM1296 (wild); OAM1297 (*efp*), *yxbC*-FLAG, OAM1306 (wild); OAM1307 (*efp*), *cydB*-FLAG, OAM1298 (wild); OAM1298 (*efp*), *yjcD*-FLAG, OAM1300 (wild); OAM1301 (*efp*), *oppC*-FLAG, OAM1302 (wild); OAM1303 (*efp*), *lctP*-FLAG, OAM1304 (wild); OAM1305 (*efp*). Except for *yfmS* and *rpoE*, the analyzed proteins carry the XPPX motif.

Table S3. Strains and plasmids used in this study.

Strain	Genotype	Reference or source
168	*trpC2*	Laboratory stock
OAM1226	*trpC2 efp*::Tn (Km^{r})	This study
OAM816	*trpC2 ylxR*::(Km^{r})	3
OAM1227	*trpC2 efp* (Km^{r}) *amyE*::Pxyl-*efp* (Cm^{r})	This study
OAM1228	*trpC2 efp* (Km^{r}) Pspac-*trxA* (Cm^{r})	This study
OAM1033	*trpC2 amyE*::Pxyl-*mntG* (Cm^{r})	4
OAM1229	*trpC2 efp* (Km^{r}) *amyE*::Pxyl-*mntG* (Cm^{r})	This study
OAM1230	*trpC2 amyE*::Pxyl-*rpoBC* (Cm^{r})	This study
OAM709	*trpC2 thrC*::*sigX-lacZ* (Em^{r})	5
OAM1231	*trpC2 thrC*::*sigX-lacZ* (Em^{r}) *efp* (Km^{r})	This study
OAM1232	*trpC2 thrC*::*sigX-lacZ* (Em^{r}) *efp* (Km^{r}) *amyE*::Pxyl-*efp* (Cm^{r})	This study
OAM1233	*trpC2 thrC*::*sigX-lacZ* (Em^{r}) *amyE*::Pxyl-*rpoBC* (Cm^{r})	This study
OAM1234	*trpC2 thrC*::*sigX-lacZ* (Em^{r}) *efp* (Km^{r}) *amyE*::Pxyl-*rpoBC* (Cm^{r})	This study
OAM1235	*trpC2 sacA*::*sigM-lacZ* (Em^{r})	This study
OAM1236	*trpC2 sacA*::*sigM-lacZ* (Em^{r}) *efp* (Km^{r})	This study
OAM1237	*trpC2 sacA*::*sigM-lacZ* (Em^{r}) *efp* (Km^{r}) *amyE*::Pxyl-*efp* (Cm^{r})	This study
OAM1238	*trpC2 sacA*::*sigM-lacZ* (Em^{r}) *amyE*::Pxyl-*rpoBC* (Cm^{r})	This study
OAM1239	*trpC2 sacA*::*sigM-lacZ* (Em^{r}) *efp* (Km^{r}) *amyE*::Pxyl-*rpoBC* (Cm^{r})	This study
MBS78	*trpC2 thrC*::*sigI-lacZ* (Em^{r})	6
OAM1240	*trpC2 thrC*::*sigI-lacZ* (Em^{r}) *ylxR* (Km^{r})	This study
OAM1242	*trpC2 thrC*::*sigI-lacZ* (Em^{r}) *efp* (Km^{r})	This study
BSU32	*trpC2 amyE*::*sigV-lacZ* (Cm^{r})	7
OAM1243	*trpC2 amyE*::*sigV-lacZ* (Cm^{r}) *ylxR* (Km^{r})	This study
OAM1244	*trpC2 amyE*::*sigV-lacZ* (Cm^{r}) *efp* (Km^{r})	This study
BSU42	*trpC2 amyE*::*sigW-lacZ* (Cm^{r})	8
OAM1245	*trpC2 amyE*::*sigW-lacZ* (Cm^{r}) *ylxR* (Km^{r})	This study
OAM1246	*trpC2 amyE*::*sigW-lacZ* (Cm^{r}) *efp* (Km^{r})	This study
BSU35	*trpC2 amyE*::*sigY-lacZ* (Cm^{r})	7
OAM1247	*trpC2 amyE*::*sigY-lacZ* (Cm^{r}) *ylxR* (Km^{r})	This study
OAM1248	*trpC2 amyE*::*sigY-lacZ* (Cm^{r}) *efp* (Km^{r})	This study
BSU36	*trpC2 amyE*::*sigZ-lacZ* (Cm^{r})	7
OAM1249	*trpC2 amyE*::*sigZ-lacZ* (Cm^{r}) *ylxR* (Km^{r})	This study
1250	*trpC2 amyE*::*sigZ-lacZ* (Cm^{r}) *efp* (Km^{r})	This study
ASK2102	*trpC2 rpoC*- 6xHis (Em^{r}) *sigB* (Cm^{r}) *sigH* (Km^{r}) *sigW* (Sp^{r})	9
OAM1251	*trpC2 rpoC*- 6xHis (Em^{r})	This study
OAM1252	*trpC2 rpoC*- 6xHis (Em^{r}) *ylxR* (Km^{r})	This study
OAM1253	*trpC2 rpoC*- 6xHis (Em^{r}) *efp* (Km^{r})	This study
OAM1254	*trpC2 sigB*- FLAG (Cm^{r})	This study
OAM1255	*trpC2 sigB*- FLAG (Cm^{r}) *efp* (Km^{r})	This study
OAM1256	*trpC2 sigD*- FLAG (Cm^{r})	This study
OAM1257	*trpC2 sigD*- FLAG (Cm^{r}) *efp* (Km^{r})	This study
OAM1258	*trpC2 fliY*- FLAG (Cm^{r})	This study
OAM1259	*trpC2 fliY*- FLAG (Cm^{r}) *efp* (Km^{r})	This study
OAM1260	*trpC2 fliW*- FLAG (Cm^{r})	This study
OAM1261	*trpC2 fliW*- FLAG (Cm^{r}) *efp* (Km^{r})	This study
OAM1262	*trpC2 motA*- FLAG (Cm^{r})	This study
OAM1263	*trpC2 motA*- FLAG (Cm^{r}) *efp* (Km^{r})	This study
OAM1264	*trpC2 motB*- FLAG (Cm^{r})	This study
OAM1265	*trpC2 motB*- FLAG (Cm^{r}) *efp* (Km^{r})	This study
OAM1266	*trpC2 trxA*- FLAG (Cm^{r})	This study
OAM1267	*trpC2 trxA*- FLAG (Cm^{r}) *efp* (Km^{r})	This study
OAM1268	*trpC2 mntG*- FLAG (Cm^{r})	This study
OAM1269	*trpC2 mntG*- FLAG (Cm^{r}) *efp* (Km^{r})	This study
OAM1270	*trpC2 mntB*- FLAG (Cm^{r})	This study
OAM1271	*trpC2 mntB*- FLAG (Cm^{r}) *efp* (Km^{r})	This study
OAM1272	*trpC2 yfmS*- FLAG (Cm^{r})	This study
OAM1273	*trpC2 yfmS*- FLAG (Cm^{r}) *efp* (Km^{r})	This study
OAM1274	*trpC2 yvmA*- FLAG (Cm^{r})	This study
OAM1275	*trpC2 yvmA*- FLAG (Cm^{r}) *efp* (Km^{r})	This study
OAM1276	*trpC2 cypX*- FLAG (Cm^{r})	This study
OAM1277	*trpC2 cypX*- FLAG (Cm^{r}) *efp* (Km^{r})	This study
OAM1278	*trpC2 yuzD*- FLAG (Cm^{r})	This study
OAM1279	*trpC2 yuzD*- FLAG (Cm^{r}) *efp* (Km^{r})	This study
OAM1280	*trpC2 narH*- FLAG (Cm^{r})	This study
OAM1281	*trpC2 narH*- FLAG (Cm^{r}) *efp* (Km^{r})	This study
OAM1282	*trpC2 albF*- FLAG (Cm^{r})	This study
OAM1283	*trpC2 albF*- FLAG (Cm^{r}) *efp* (Km^{r})	This study
OAM1284	*trpC2 pnbA*- FLAG (Cm^{r})	This study
OAM1285	*trpC2 pnbA*- FLAG (Cm^{r}) *efp* (Km^{r})	This study
OAM1286	*trpC2 yvcK*- FLAG (Cm^{r})	This study
OAM1287	*trpC2 yvcK*- FLAG (Cm^{r}) *efp* (Km^{r})	This study
OAM1288	*trpC2 yscB*- FLAG (Cm^{r})	This study
OAM1289	*trpC2 yscB*- FLAG (Cm^{r}) *efp* (Km^{r})	This study
OAM1290	*trpC2 nasF*- FLAG (Cm^{r})	This study
OAM1291	*trpC2 nasF*- FLAG (Cm^{r}) *efp* (Km^{r})	This study
OAM1292	*trpC2 rpoE*- FLAG (Cm^{r})	This study

OAM1293	*trpC2 rpoE*- FLAG (Cm^r) *efp* (Km^r)	This study
OAM1294	*trpC2 csaA*- FLAG (Cm^r)	This study
OAM1295	*trpC2 csaA*- FLAG (Cm^r) *efp* (Km^r)	This study
OAM1296	*trpC2 defB*- FLAG (Cm^r)	This study
OAM1297	*trpC2 defB*- FLAG (Cm^r) *efp* (Km^r)	This study
OAM1306	*trpC2 yxbC*- FLAG (Cm^r)	This study
OAM1307	*trpC2 yxbC*- FLAG (Cm^r) *efp* (Km^r)	This study
OAM1298	*trpC2 cydB*- FLAG (Cm^r)	This study
OAM1299	*trpC2 cydB*- FLAG (Cm^r) *efp* (Km^r)	This study
OAM1300	*trpC2 yjcD*- FLAG (Cm^r)	This study
OAM1301	*trpC2 yjcD*- FLAG (Cm^r) *efp* (Km^r)	This study
OAM1302	*trpC2 oppC*- FLAG (Cm^r)	This study
OAM1303	*trpC2 oppC*- FLAG (Cm^r) *efp* (Km^r)	This study
OAM1304	*trpC2 lctP*- FLAG (Cm^r)	This study
OAM1305	*trpC2 lctP*- FLAG (Cm^r) *efp* (Km^r)	This study
Plasmid	Description	
pX	Amp^r *amyE* ::*xylR* -Pxyl Cm^r	10
pX-efp	Px carrying *efp* (*efp* ORF with its SD), Cm^r	This study
pYY1	Px carrying *rpoBC* (*rpoBC* ORFs with their SD), Cm^r	Kosono S
pMutinIII-His	Insertion vecter, ampicillin and erythromycin resistance, *lacZI*	This study
pMutin-H-trxA	pMutinIII-His carrying a N-terminal part of *the trxA* ORF	This study
pSac-Km	Amp^r Km^r *sacA*	1
pDG1663	Amp^r Tc^r Em^r *thrC*	11
pMT6	pDG1663 derivative carrying *thrC*::*sigM-lacZ* (Em^r)	2
pSac-Em-sigM	Amp^r Tc^r Em^r *sacA* ::*sigM* -*lacZ*	Asai K
pCA3xFLAG	Amp^r, FLAG, Cm^r	12
pflag-sigB	pCA3xFLAG carrying C-terminal region of *sigB*	This study
pflag-sigD	pCA3xFLAG carrying C-terminal region of *sigD*	This study
pflag-fliY	pCA3xFLAG carrying C-terminal region of *fliY*	This study
pflag-fliW	pCA3xFLAG carrying C-terminal region of *fliW*	This study
pflag-motA	pCA3xFLAG carrying C-terminal region of *motA*	This study
pflag-motB	pCA3xFLAG carrying C-terminal region of *motB*	This study
pflag-trxA	pCA3xFLAG carrying C-terminal region of *trxA*	This study
pflag-mntG	pCA3xFLAG carrying C-terminal region of *mntG*	This study
pflag-mntB	pCA3xFLAG carrying C-terminal region of *mntB*	This study
pflag-yfmS	pCA3xFLAG carrying C-terminal region of *yfmS*	This study
pflag-yvmA	pCA3xFLAG carrying C-terminal region of *yvmA*	This study
pflag-cypX	pCA3xFLAG carrying C-terminal region of *cypX*	This study
pflag-yuzD	pCA3xFLAG carrying C-terminal region of *yuzD*	This study
pflag-narH	pCA3xFLAG carrying C-terminal region of *narH*	This study
pflag-albF	pCA3xFLAG carrying C-terminal region of *albF*	This study
pflag-pnbA	pCA3xFLAG carrying C-terminal region of *pnbA*	This study
pflag-yvcK	pCA3xFLAG carrying C-terminal region of *yvcK*	This study
pflag-yscB	pCA3xFLAG carrying C-terminal region of *yscB*	This study
pflag-nasF	pCA3xFLAG carrying C-terminal region of *nasF*	This study
pflag-rpoE	pCA3xFLAG carrying C-terminal region of *rpoE*	This study
pflag-csaA	pCA3xFLAG carrying C-terminal region of *csaA*	This study
pflag-defB	pCA3xFLAG carrying C-terminal region of *defB*	This study
pflag-yxbC	pCA3xFLAG carrying C-terminal region of *yxbC*	This study
pflag-cydB	pCA3xFLAG carrying C-terminal region of *cydB*	This study
pflag-yjcD	pCA3xFLAG carrying C-terminal region of *yjcD*	This study
pflag-oppC	pCA3xFLAG carrying C-terminal region of *oppC*	This study
pflag-lctP	pCA3xFLAG carrying C-terminal region of *lctP*	This study

Table S4. Oligonucleotide sequences for construction of the plasmids.

Oligo name	Sequence	Product	Vector	Restriction enzymes
pX-efp-Spe	5-AAACTAGTGGAATATAGGAGGACATTAAAC-3	pX-efp	pX	SpeI/BamHI
pX-efp-Bam	5-ATGGGATCCTATGCTCTTGAAACGTAAGA-3	pX-efp	pX	SpeI/BamHI
pXrpoBCinfus_f	5-AATGGTCCAAACTAGAAAGGAGGATACTCAGATGACAGGTCAACTAG-3	pYY1	pX	Not use
pXrpoBCinfus_r	5-TTTAGATATCACTAGTTATTCAACCGGGACCAT-3	pYY1	pX	Not use
pMut-His-trx-Sal	5-ATGGTCGACATTTCACATTGGAGGAATTC-3	pMutin-H-trxA	pMutinIII-His	SalI/XhoI
pMut-His-trx-Xh	5-GCGCTCGAGCGCTTCTTTTGGTTTGAAGCCG-3	pMutin-H-trxA	pMutinIII-His	SalI/XhoI
Pflag-sigB-H	5-TTGAAGCTTTTGCAATCCCG-3	pflag-sigB	pCA3xFLAG	HindIIII/XbaI
Pflag-sigB-R-Xb	5-GATCTAGACATTAACTCCATCGAGGGA-3	pflag-sigB	pCA3xFLAG	HindIIII/XbaI
Pflag-sigD-F-Bg	5-TTCAGATCTGTCGGACTGCCGAAATC-3	pflag-sigD	pCA3xFLAG	BamHI/BglII/XbaI
Pflag-sigD-R-Xb	5-GATCTAGATTGTATCACTTTTTCCAGCAGA-3	pflag-sigD	pCA3xFLAG	BamHI/BglII/XbaI
FLAG-fliY-E	5-CCGGAATTCAAGCCTTATGAACTCAGAAAG-3	pflag-fliY	pCA3xFLAG	EcoRI/XbaI
FLAG-fliY-Xb	5-GATCTAGATTTTAAATTATTAATGCGCTCTGC-3	pflag-fliY	pCA3xFLAG	EcoRI/XbaI
FLAG-fliW-E	5-CCGGAATTCATACGAAGTACCATGGCC-3	pflag-fliW	pCA3xFLAG	EcoRI/XbaI
FLAG-fliW-Xb	5-CGATCTAGAGCATGATTCTCCTCCAATCGG-3	pflag-fliW	pCA3xFLAG	EcoRI/XbaI
FLAG-motA-E	5-CCGGAATTCTTGAAAAACGGGCTCAGC-3	pflag-motA	pCA3xFLAG	EcoRI/XbaI
FLAG-motA-Xb	5-CGATCTAGATGCTTCTTCTTCTTTTTTCTCGCC-3	pflag-motA	pCA3xFLAG	EcoRI/XbaI
FLAG-motB-H	5-CCGAAGCTTACAGGCGGAACCGGTGTA-3	pflag-motB	pCA3xFLAG	HindIIII/XbaI
FLAG-motB-Xb	5-CGATCTAGATTTTTCATTTGTTTCCGCTGCGC-3	pflag-motB	pCA3xFLAG	HindIIII/XbaI
FLAG-trx-E	5-GAGGAATTCAATAATGGCTATCG-3	pflag-trxA	pCA3xFLAG	EcoRI/XbaI
FLAG-trx-Xb	5-GATCTAGAAAGATGTTTGTTTACAAGCTCTTG-3	pflag-trxA	pCA3xFLAG	EcoRI/XbaI
FLAG-yscB-E	5-CCGGAATTCGCCGCTGAGAAAAAGCT-3	pflag-mntG	pCA3xFLAG	EcoRI/XbaI
FLAG-yscB-Xb	5-GATCTAGATGGTGCGTCTTCGGGATCG-3	pflag-mntG	pCA3xFLAG	EcoRI/XbaI
FLAG-mntB-E	5-CCGGAATTCGGGATGCATGATTATGCG-3	pflag-mntB	pCA3xFLAG	EcoRI/XbaI
FLAG-mntB-Xb	5-GATCTAGACTCCTTATGTCCTTCTGCCA-3	pflag-mntB	pCA3xFLAG	EcoRI/XbaI
FLAG-yfmS-E	5-CCGGAATTCGCCGATATTTCAGTAACTGT-3	pflag-yfmS	pCA3xFLAG	EcoRI/XbaI
FLAG-yfmS-Xb	5-GATCTAGACTCTTCCTCAAGCGCTTTTTC-3	pflag-yfmS	pCA3xFLAG	EcoRI/XbaI
FLAG-yvmA-E	5-CCGGAATTCGGAAGCAATGTGATTGCCG-3	pflag-yvmA	pCA3xFLAG	EcoRI/XbaI
FLAG-yvmA-Xb	5-GATCTAGACATGTGGTGCTTTTTTTGTTTCG-3	pflag-yvmA	pCA3xFLAG	EcoRI/XbaI
FLAG-cypX-E	5-CCGGAATTCCGAGAGCCATT GCGG-3	pflag-cypX	pCA3xFLAG	EcoRI/XbaI
FLAG-cypX-Xb	5-GATCTAGATGCCCCGTCAAACGCAAC-3	pflag-cypX	pCA3xFLAG	EcoRI/XbaI
FLAG-yuzD-E	5-CCGGAATTCCAGTCATGCTAAGCGTGTAC-3	pflag-yuzD	pCA3xFLAG	EcoRI/XbaI
FLAG-yuzD-Xb	5-GATCTAGAGCGGTTTTCTGTATACCCATGC-3	pflag-yuzD	pCA3xFLAG	EcoRI/XbaI
FLAG-narH-E	5-CCGGAATTCCGCCGCTCAGCCCG-3	pflag-narH	pCA3xFLAG	EcoRI/XbaI
FLAG-narH-Xb	5-GATCTAGAGAAACAGGAGCCGGGGCC-3	pflag-narH	pCA3xFLAG	EcoRI/XbaI
FLAG-albF-E	5-CCGGAATTCTATTTCAATTAGATTCACCG-3	pflag-albF	pCA3xFLAG	EcoRI/XbaI
FLAG-albF-Xb	5-GATCTAGATTGTCTGATCGCCTCTTTC-3	pflag-albF	pCA3xFLAG	EcoRI/XbaI
FLAG-pnbA-E	5-CCGGAATTCCGCGTTCTCTGGAAAGC-3	pflag-pnbA	pCA3xFLAG	EcoRI/XbaI
FLAG-pnbA-Xb	5-GATCTAGATTCTCCTTTTGAAGGGAATAG-3	pflag-pnbA	pCA3xFLAG	EcoRI/XbaI
FLAG-yvcK-E	5-CCGGAATTCACGCCTGAACAGATTGATCC-3	pflag-yvcK	pCA3xFLAG	EcoRI/XbaI
FLAG-yvcK-Xb	5-GATCTAGATTCTTTCAGTAAATCAACAAGAAGAG-3	pflag-yvcK	pCA3xFLAG	EcoRI/XbaI
FLAG-yscB-E	5-CCGGAATTCGCCGCTGAGAAAAAGCT-3	pflag-yscB	pCA3xFLAG	EcoRI/XbaI
FLAG-yscB-Xb	5-GATCTAGATGGTGCGTCTTCGGGATCG-3	pflag-yscB	pCA3xFLAG	EcoRI/XbaI
FLAG-nasF-E	5-CCGGAATTCAGAGAAACGTCTTGTTGTC-3	pflag-nasF	pCA3xFLAG	EcoRI/XbaI
FLAG-nasF-Xb	5-GATCTAGAGGTGTGAAGGAGTTTTTTCTCT-3	pflag-nasF	pCA3xFLAG	EcoRI/XbaI
FLAG-rpoE-E	5-CCGGAATTCTTGGAGACCGCATTGCTCA-3	pflag-rpoE	pCA3xFLAG	EcoRI/XbaI
FLAG-rpoE-Xb	5-GATCTAGATTTAATTTCCTCTTCTTCATCATC-3	pflag-rpoE	pCA3xFLAG	EcoRI/XbaI
FLAG-csaA-E	5-CCGGAATTCGGCAGTTATTGATGACTTTGAG-3	pflag-csaA	pCA3xFLAG	EcoRI/XbaI
FLAG-csaA-Xb	5-GATCTAGATCCGATTTTTGTGCCGTTTGGG-3	pflag-csaA	pCA3xFLAG	EcoRI/XbaI
FLAG-defB-E	5-CCGGAATTCAGAGAGACTGCCGAACC-3	pflag-defB	pCA3xFLAG	EcoRI/XbaI
FLAG-defB-Xb	5-GATCTAGAGCGCTCAATTGCGATTGCAT-3	pflag-defB	pCA3xFLAG	EcoRI/XbaI
FLAG-yxbC-E	5-CCGGAATTCATATTGGAAAGGAGATCCGC-3	pflag-yxbC	pCA3xFLAG	EcoRI/XbaI
FLAG-yxbC-Xb	5-CGATCTAGAGAACTTATAACTTGTCAAAAGCTG-3	pflag-yxbC	pCA3xFLAG	EcoRI/XbaI
FLAG-cydB-E	5-CCGGAATTCTTGCCTTCGCGGCC-3	pflag-cydB	pCA3xFLAG	EcoRI/XbaI
FLAG-cydB-Xb	5-CGATCTAGAATAAGTCATAGGCTCCTTATGGC-3	pflag-cydB	pCA3xFLAG	EcoRI/XbaI
FLAG-yjcD-H	5-CCGAAGCTTCTCCGAGTATTTAAAAAAGCGG-3	pflag-yjcD	pCA3xFLAG	HindIIII/XbaI
FLAG-yjcD-Xb	5-CGATCTAGAATGATGCAAGGGCTGTCTTGCTTTC-3	pflag-yjcD	pCA3xFLAG	HindIIII/XbaI
FLAG-oppC-H	5-CCGAAGCTTCCGAAAGGTCTATTTACGA-3	pflag-oppC	pCA3xFLAG	HindIIII/XbaI
FLAG-oppC-Xb	5-CGATCTAGATTTACGTAACTTAGGATCCAATGC-3	pflag-oppC	pCA3xFLAG	HindIIII/XbaI
FLAG-lctP-E	5-CCGGAATTCAGCAAAAAGTTCTCCTCTCG-3	pflag-lctP	pCA3xFLAG	EcoRI/XbaI
FLAG-lctP-Xb	5-CGATCTAGACACTCCTAATCTTGCAAGAATAAAC-3	pflag-lctP	pCA3xFLAG	EcoRI/XbaI

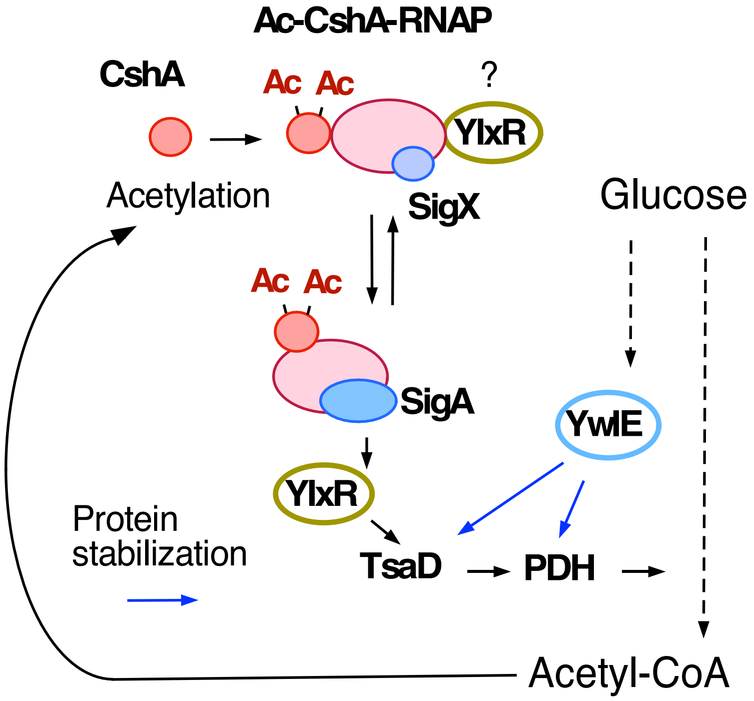
Ac-CshA-RNAP
CshA
Ac
Ac
?
YlxR
Acetylation
SigX
Glucose
Ac
Ac
SigA
YwlE
YlxR
Protein
stabilization
TsaD
PDH
Acetyl-CoA

Fig. S1

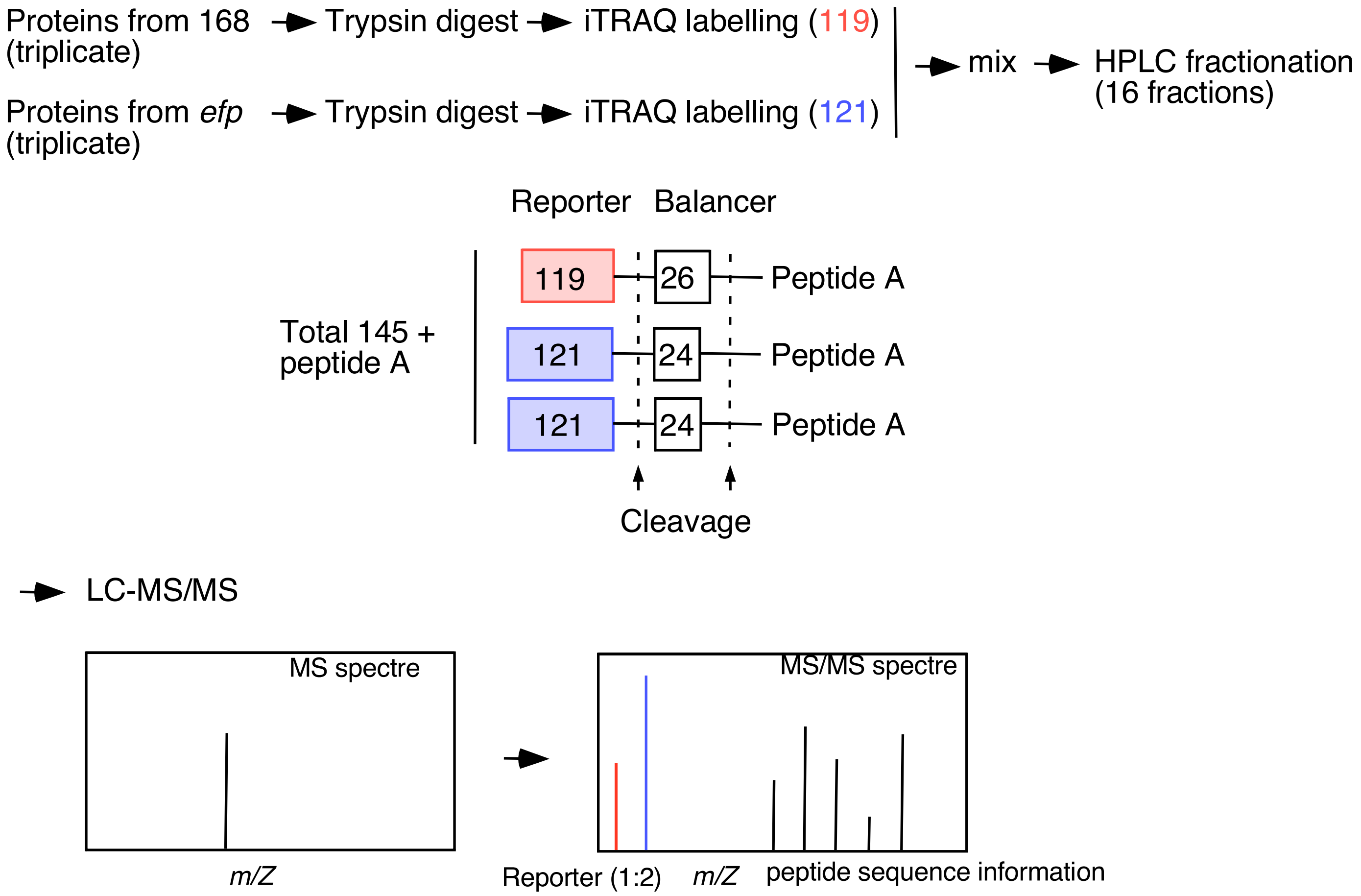
Proteins from 168
(triplicate)
Trypsin digest
iTRAQ labelling (119)
Proteins from *efp*
(triplicate)
Trypsin digest
iTRAQ labelling (121)
mix
HPLC fractionation
(16 fractions)
Reporter
Balancer
Total 145 +
peptide A
119
26
Peptide A
121
24
Peptide A
121
24
Peptide A
Cleavage
LC-MS/MS
MS spectre
m/Z
MS/MS spectre
Reporter (1:2)
m/Z
peptide sequence information

Fig. S2

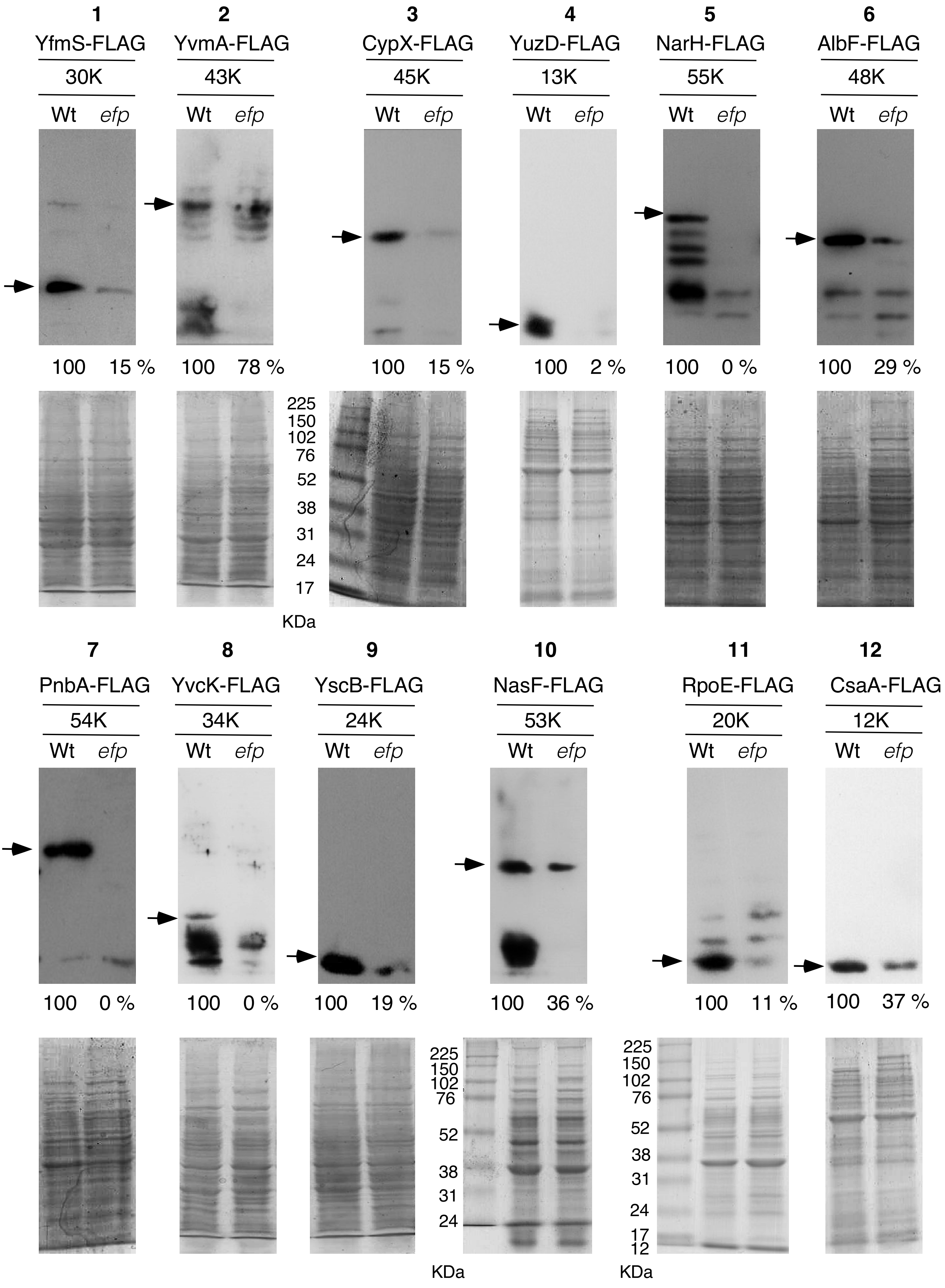
1
YfmS-FLAG
30K
Wt efp
100 15 %
2
YvmA-FLAG
43K
Wt efp
100 78 %
3
CypX-FLAG
45K
Wt efp
100 15 %
225
150
102
76
52
38
31
24
17
KDa
4
YuzD-FLAG
13K
Wt efp
100 2 %
5
NarH-FLAG
55K
Wt efp
100 0 %
6
AlbF-FLAG
48K
Wt efp
100 29 %
7
PnbA-FLAG
54K
Wt efp
100 0 %
8
YvcK-FLAG
34K
Wt efp
100 0 %
9
YscB-FLAG
24K
Wt efp
100 19 %
10
NasF-FLAG
53K
Wt efp
100 36 %
225
150
102
76
52
38
31
24
KDa
11
RpoE-FLAG
20K
Wt efp
100 11 %
225
150
102
76
52
38
31
24
17
12
KDa
12
CsaA-FLAG
12K
Wt efp
100 37 %

Fig. S3A

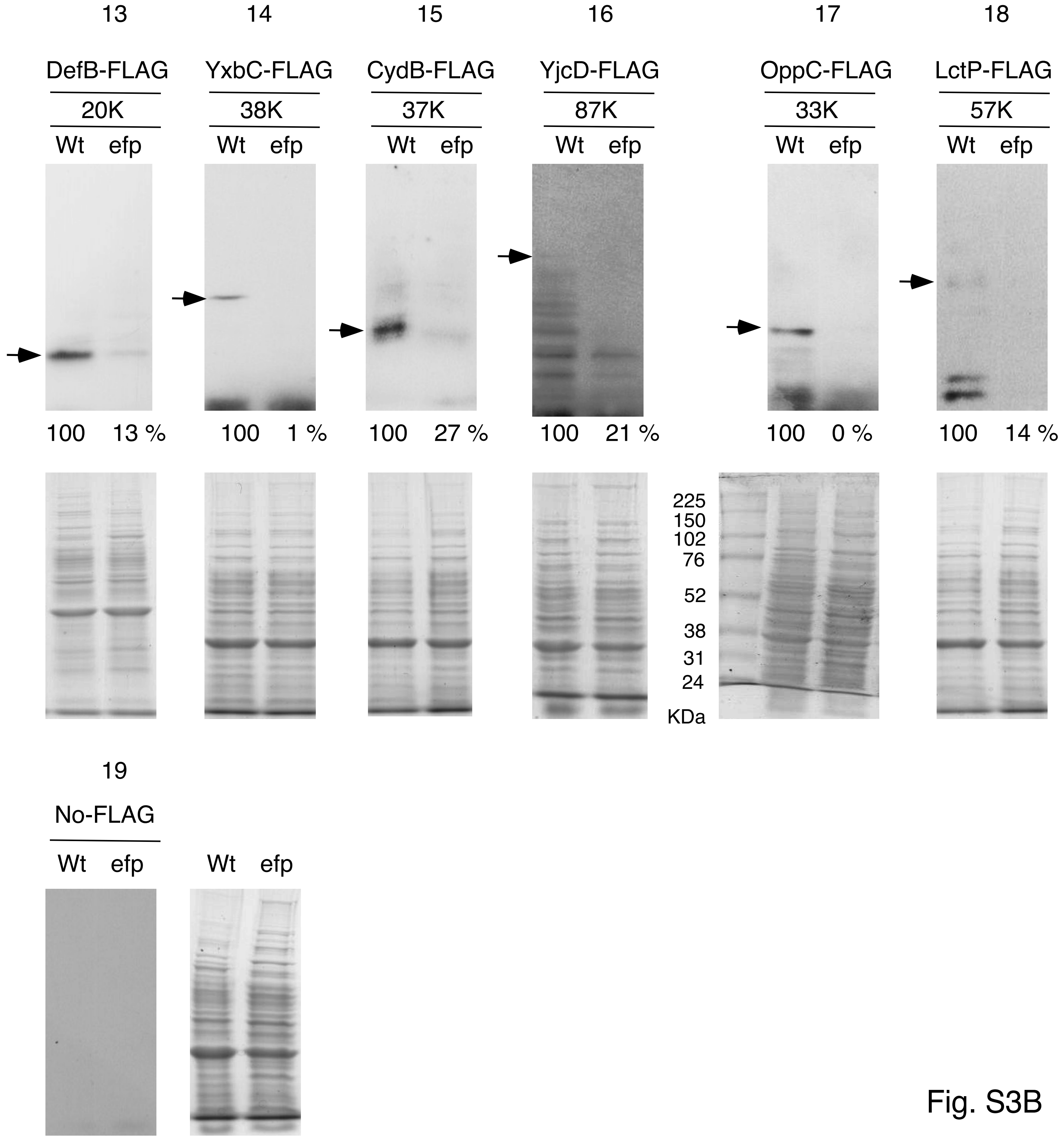
13
DefB-FLAG
20K
Wt efp
100 13 %
14
YxbC-FLAG
38K
Wt efp
100 1 %
15
CydB-FLAG
37K
Wt efp
100 27 %
16
YjcD-FLAG
87K
Wt efp
100 21 %
17
OppC-FLAG
33K
Wt efp
100 0 %
18
LctP-FLAG
57K
Wt efp
100 14 %
225
150
102
76
52
38
31
24
KDa
19
No-FLAG
Wt efp
Wt efp

Fig. S3B
